# Social determinants of health and dual sensory loss in older adults: A scoping review

**DOI:** 10.1371/journal.pone.0338322

**Published:** 2025-12-10

**Authors:** Abinethaa Paramasivam, Ved Hatolkar, Renu Minhas, Aswen Sriranganathan, Pineal Bareamichael, Atul Jaiswal, Walter Wittich

**Affiliations:** 1 Department of International Health, Johns Hopkins Bloomberg School of Public Health, Baltimore, Maryland, United States of America; 2 Temerty Faculty of Medicine, University of Toronto, Toronto, Ontario, Canada; 3 DeafBlind Ontario Services, Newmarket, Ontario, Canada; 4 Faculty of Medicine, University of Ottawa, Ottawa, Ontario, Canada; 5 Department of Health Policy and Management, Johns Hopkins Bloomberg School of Public Health, Baltimore, Maryland, United States of America; 6 Perley Health, Ottawa, Ontario, Canada; 7 Interdisciplinary School of Health Sciences, University of Ottawa, Ottawa, Ontario, Canada; 8 School of Optometry, Université de Montréal, Montréal, Québec, Canada; Kafkas University: Kafkas Universitesi, TÜRKIYE

## Abstract

**Background:**

Social determinants of health (SDH), such as socioeconomic status, education, healthcare access, and social support, play a significant role in shaping individuals lived experiences. Dual sensory loss (DSL), a distinct disability involving both vision and hearing loss, poses greater challenges for daily living compared to the general population. This scoping review synthesized evidence on how various SDH indicators influence the life experiences of older adults with DSL.

**Methods:**

Five scientific databases were searched from January 2014 to May 2024. Articles focusing on individuals aged 60 and older with DSL, in the context of at least one SDH indicator, were included.

**Results:**

A total of 69 studies met the eligibility criteria. Most studies addressed the following SDH indicators: disability (n = 46), social inclusion and non-discrimination (n = 21), gender (n = 10), and access to healthcare services (n = 9). Disability-related indicators revealed higher risks of mobility limitations, cognitive decline, depression, anxiety, social isolation, and workplace discrimination, all adversely affecting mental health and quality of life. Older adults with DSL encounter significant barriers to accessing healthcare, such as absence of adequate assistive devices, communication challenges, and high healthcare costs. Many report dissatisfaction with the quality of care received.

**Conclusions:**

Our review identifies disparities that increase the vulnerability of older adults with DSL and restrict their access to healthcare, rehabilitation, and social participation. These findings warrant further research on underexplored SDH factors, using robust data sources that collects information on the lived experiences of older adults with DSL. Addressing these social determinants requires a comprehensive approach, including raising awareness, improving service access, enhancing social support networks, and ensuring inclusive policies and practices.

## Introduction

The United Nations Convention on the Rights of Persons with Disabilities highlights the systemic challenges people with disabilities face in realizing their full potential [1]. The World Health Organization (WHO) defines social determinants of health (SDH) as non-medical factors that shape health outcomes [2]. These include the conditions in which individuals are born, grow, work, live, and age, as well as the broader forces and systems influencing everyday life, such as economic policies, social norms, and systems [1]. Specifically, the WHO identifies ten SDH, which are: income and social protection, education, unemployment and job security, working life conditions, food insecurity, housing, basic amenities, and the environment, early childhood development, social inclusion and non-discrimination, structural conflict, and access to affordable, quality health services [3]. In countries such as the USA, New Zealand, Australia, Nordic nations, and Canada, three additional SDH – Indigenous status, disability, and gender [3–10] are recognized.

Dual sensory loss (DSL) is a distinct disability characterized by varying degrees of hearing and vision loss, which significantly impact communication, access to information, and mobility [11,12]. DSL can be classified into three types: congenital, acquired, and age-related [13]. Individuals with DSL make up approximately 0.2–2% of the global population [14]. While many older adults with DSL experience age-related losses, some have congenital or acquired DSL. This review does not differentiate between these heterogeneous groups within the older adult DSL population.

Older adults with DSL face substantial and often overlooked effects on their health and well-being, employment, social inclusion, mobility, independence, and access to services [14,15]. A 2018 report by the World Federation of the Deafblind found that individuals with DSL experience greater SDH inequalities compared to those with other disabilities, particularly in areas such as poverty, social inclusion, employment, socioeconomic status, gender, health, access to healthcare, and education [11]. Simcock and Wittich (2019) argue that older adults with DSL are not prioritized despite the implementation of the UN Principles for Older Persons [15]. Specifically, older adults with DSL face barriers such as insufficient interpreter-guided support, limited employment and community participation opportunities, and inadequate access to healthcare [15]. However, few studies have examined the causal effects of SDH on older adults with DSL.

In international aging health policy, the age at which individuals are considered “older adults” is typically 60 years and above, though this can vary slightly depending on the organization or region. In many low- and middle-income countries (LMICs), life expectancy is lower, meaning 60 is a more appropriate threshold for ageing-related services. In high-income countries (HIC) adults are considered “older adults” starting at age 65 and this classification is widely used in healthcare, social services, and demographic research. The United Nations uses 60+ as the standard threshold for defining “older persons” in global policy frameworks. As well, the World Health Organization (WHO) defines older adults as those aged 60, emphasizing that chronological age is not a precise marker of aging-related changes, and policies should consider functional ability and health status.

As a result, this review aims to synthesize the available evidence on how various SDH indicators influence the experiences of older adults with DSL. By doing so, we seek to identify knowledge gaps and suggest future research directions to address SDH critical to the issues of older adults with DSL. Ultimately, this review will enhance the visibility of older adults with DSL as a priority among policymakers and healthcare professionals, helping them to address these factors and improve the quality of life of this marginalized population.

## Methods

This scoping review was conducted using Arksey and O’Malley’s methodological framework, which is guided by five stages: [1] identifying the research question, [2] identifying relevant studies, [3] selecting studies, [4] charting the data, and [5] collating, summarizing, and reporting the results [16]. The review incorporated the definition of population, concept, and context as recommended by the Joanna Briggs Institute Manual for Evidence Synthesis [17]. It also adhered to the Preferred Reporting Items for Systematic Reviews and Meta-Analyses (PRISMA) extension for scoping reviews [18]. Finally, Braun and Clarke’s principles of thematic analysis were applied to analyze the findings.

### Identifying the research question

This review was guided by the research question: *What are the key social determinants of health identified in existing literature that significantly affect the experiences of older adults with age-related dual sensory loss (hearing and vision loss)?* These determinants include factors related to socioeconomic status, access to healthcare, education level, community support, and environmental conditions. According to the United Nations, older adults are individuals aged 60 years and older. For this review, older adults with DSL are characterized as those experiencing a combined loss of hearing and vision so severe that neither sense can effectively access information or facilitate community participation [13].

We examined a diverse population of older adults with DSL stemming from acquired, congenital, and age-related causes. This review investigated a total of 13 social determinants of health (SDH), comprising the 10 SDH identified by the World Health Organization (WHO), along with 3 additional SDH recognized by the American, Australian, Nordic, New Zealandia, and Canadian governments. Given the broad scope of disability as an SDH, we focused specifically on DSL and sometimes other comorbidities such as depression and dementia affecting older adults.

### Identifying the relevant studies

The research team developed a search strategy in collaboration with a health science librarian at Toronto Metropolitan University. Searches were conducted in the MEDLINE, EMBASE, CINAHL, Global Health, and PsycINFO databased, covering the period from January 2014 to May 2024 to focus on the most recent research activity on the topic. The process adhered to the guidelines of the Preferred Reporting Items for Systematic Review and Meta-Analyses (PRISMA) [19]. For checklist see S1 File PRISMA Scr Checklist (S1 File) The search results were imported into reference management software, Mendeley Desktop Version 1.19.6 (Mendeley, London, United Kingdom) and subsequently transferred to Covidence, a screening tool, for abstract and full-text screening.

### Study selection

Five members of the research team (A.P., V.H., A.J., and P.B.) reviewed and screened articles at the title/abstract level and full text-level based on the established eligibility criteria (Table 1). When there was disagreement with the inclusion of an article, more senior team members (R.M. or A.J.) resolved conflicts. Selected articles were recorded on a Microsoft Excel 2020 spreadsheet for data extraction.

**Table 1:** Eligibility criteria for studies included in this scoping review.

Inclusion Criteria	Exclusion Criteria
At minimum 5 study participants who are older adults aged 60+ with DSL	Study participants are older adults with single sensory impairment (only hearing impairment or vision impairment).
Studies focus on at least one of the SDH	Studies that focus on genetics, psychometric, preventative, phase II clinical trials, and/or laboratory experiments involving animals.
Randomized control trials, clinical controlled trials, cross-sectional studies, observational studies, and qualitative studies.	Literature reviews, retrospective reviews, study protocols, letters, editorials, books or book chapters and commentaries.
Studies published between January 2014 and May 2024	

### Charting the data

The research team reviewed the selected articles and developed a data charting template using Microsoft Excel 2020 following a group consultation. The charting template contained descriptors like the author’s name, year of study, country of study, study population, methods of data collection, number of participants, gender of participants, sociodemographic characteristics, type of DSL, the type of SDH covered, affecting older adults with DSL can be found in supporting information (S2 File). Authors A.P., V.H., A.S., and P.B. reviewed and extracted the data using the descriptors in the spreadsheet. Authors (R.M., A.J., and W.W.) provided support and/or resolved any differences in extraction.

### Collating, summarizing, and reporting the results

The results were analyzed using qualitative thematic analysis and presented in tables and figures. The research team convened to discuss data extraction and interpret findings related to older adults with DSL. They determined the most effective way to present the synthesis was by emphasizing the most frequently occurring themes and SDH identified in the literature.

## Results

### Study characteristics

Out of the 4,687 articles identified from five scholarly databases, sixty-nine studies met the eligibility criteria. (S1 Fig) illustrates the number of articles identified, screened, and deemed eligible. The studies were conducted in United States (n = 17), China (n = 13), Japan (n = 5), Australia (n = 5), Canada (n = 4), United Kingdom/South Korea/Europe/Denmark (each n = 3), Sweden/Scotland/Netherlands (each n = 2), and Norway/multi-country/Malaysia/India/Iceland/Germany/France (each n = 1). Only one study was conducted in a low-middle income country (India). From 2014 to 2024, most studies were published in 2022 (n = 15) while there was a modest number of published studies between 2018–2020 (S2 Fig) Some studies (n = 13) did not provide a breakdown of age ranges. Consequently, these studies included participants younger than 65 years, but their age range extending beyond 65 years (e.g., 45–89 years). Importantly, the studies included in this scoping review show methodological diversity, with a large presence of longitudinal designs (n = 34), constrasting with relatively fewer cross-sectional (n = 18), qualitative (n = 11), and quantitative (n = 6) studies. Furthermore, sample sizes varied greatly from small communities to large population datasets; however, inconsistencies in age reporting and demographic breakdowns limit interpretability. Notably, the literature shows strong geographic bias with all but one study originating from high income countries (HICs). This limits generalizability to low-resource settings where disparities may be more pronounced. Similarly, there is an over-representation of disability and social inclusion studies that although are informative, may overshadow underexplored determinants such as race, income, and food insecurity. Common terms used to describe DSL included deafblindness, dual sensory impairment, hearing and vision loss, sensory impairments, concurrent hearing, and vision impairment. Table 2 presents the number of studies per SDH indicator.

**Table 2:** Number of studies per SDH included in analyses.

Social Determinant of Health Indicator	Number of Studies
Disability	46
Social Inclusion and Non-Discrimination	21
Gender	10
Access to Affordable Health	9
Income and Social Protection	6
Education	6
Housing, Basic Amenities, and the Environment	5
Unemployment and Job Security	3
Race	2
Food Insecurity	1
Working Life Conditions	0
Structural Conflict	0
Indigenous Status	0

### Disability

Forty-six studies that identified the disability of older adults with DSL revealed its association with increased risks of mobility, limitations in activities of daily living, such as self-care and household activities. Furthermore, studies revealed DSL is strongly associated with cognitive decline and increased dementia. Compared to those with either hearing impairment or vision impairment alone, individuals with DSL experienced a faster rate of cognitive decline and an increased risk of developing dementia [20–22]. Additionally, DSL was linked to poorer mental health outcomes, with affected individuals demonstrating a higher prevalence of depressive symptoms, anxiety, and other psychological issues [20,23,24]. For instance, one study found that participants with DSL had a higher prevalence of cognitive impairment (50.2%), depression (53.1%), and anxiety (16.9%) compared to those with no sensory impairments or with single sensory impairment. Depression and anxiety have also been shown to mediate the relationship between DSL and other health outcomes, such as cognitive function, further exacerbating the negative impact of DSL on quality of life [23–25]. One study found that individuals with DSL had a 28.8% incidence rate of all-cause dementia over a mean follow-up period of 11.1 years, which was significantly higher compared to individuals with single sensory impairments [26].

### Social inclusion and non-discrimination

Twenty-one studies examined the impact of social inclusion and non-discrimination on older adults with DSL, revealing significant challenges in social isolation, discrimination, and psychosocial well-being. Social isolation was highly prevalent in individuals with DSL, whereby 68.6% were socially inactive with low educational and community participation [27]. DSL was associated with increased isolation over time compared to single sensory impairments [28]. Communication difficulties exacerbate this isolation, leading to withdrawal from social interactions [23,29], and reduced participation in social activities was noted among those lacking adequate sensory aids [30]. While some variability existed—with 57.7% not experiencing low social support—emotional isolation and limited social contact were still reported [31].

Workplace discrimination and forced retirement were significant concerns; individuals with DSL faced higher rates of forced retirement and changes in work due to sensory impairments [32], experienced more discrimination than those with single impairment [33], and had a retirement rate of 94.5% compared to 62.5% in those without impairments [34]. The combined effects contributed to adverse psychosocial outcomes, including depression, anxiety, cognitive decline, and increased mortality; depressive symptoms and loneliness correlated with poorer mental health-related quality of life [35] and increased rates of loneliness and depression were reported [36,37].

### Gender

Ten studies examined the association between gender (people) and DSL, collectively highlighting significant gender differences in the prevalence and impact of sensory impairments. Across multiple studies, it was observed that women generally had a higher prevalence of vision impairment (VI), while men were more frequently affected by hearing impairment (HI) and DSL [38–42]. One study found that DSL, rather than HI or VI alone, was associated with a significantly increased risk of mobility and activity of daily living difficulties in both men and women, with stronger effects noted in women females [31]. Another study revealed that men with sensory impairments exhibited a higher prevalence of cognitive impairment, depression, and anxiety compared to women [21]. Additionally, men with DSL were more prone to depression [41]. Similarly, women with DSL reported more functional limitations, physical inactivity, and lower educational status while men with DSL reported higher daily smoking [42].

Moreover, the data indicates that certain demographic factors, such as age, marital status, and education, intersected with gender to influence the prevalence and severity of sensory impairments [25,39,43]. When considering DSL that is due to Usher Syndrome Type II, men, were more likely to experience more severe impairments and related health issues such as tinnitus and high blood pressure compared to women [43]. The studies also found that older adults with DSL were predominantly women, particularly in urban and rural settings, and were more likely to be illiterate, single, and unemployed [39].

### Access to health care services

Nine studies focused on access to health care services for older adults with DSL. The studies collectively emphasized the multifaceted challenges faced by individuals with DSL and the critical need for targeted interventions across various aspects of healthcare. Several studies highlighted that, while hearing and vision impairments are often addressed separately, DSL is frequently overlooked, pointing to the need for better guidelines and resources in community pharmacies and care homes [22,30]. Barriers to accessing optimal pharmaceutical care were also identified, including communication difficulties, lack of training, and inadequate assistive technology, all of which contributed to suboptimal care [31]. The dissatisfaction with healthcare quality was notably higher among those with DSL, particularly in relation to the perceived concern of doctors about overall health and the information provided to patients [32]. Additionally, insufficient correction for sensory impairments, such as inadequate hearing aids and vision devices, was common among older women, exacerbating their health challenges [33]. Financial implications were also evident, with DSL being associated with higher healthcare costs and catastrophic health expenditures, particularly among those with additional factors like depression [25]. Despite these challenges, rehabilitation services play a vital role in helping individuals with DSL maintain their life roles, although accessibility and professional support significantly improved outcomes [44]. Finally, the existential concerns of older adults with DSL, including their desire for participation and control in care delivery, underscore the importance of addressing both physical and emotional needs in this population [45].

### Housing, basic amenities, and the environment

Five studies focused on housing, basic amenities, and the environment. The studies collectively highlighted the intersection of living conditions, geographic location, and social support with DSL and related outcomes. Individuals living alone were more likely to experience DSL, reflecting a link between isolation and sensory impairments [30,46]. A worse living standard was associated with higher rates of vision loss, hearing loss, and DSL, as well as unmet needs for assistive devices, whereas better living standards correlated with regular use of glasses and hearing aids [30]. Compared to urban dwellers, rural residents, consistently reported a higher prevalence of both DSL and loneliness, indicating geographic disparities in sensory health and social well-being [47,48]. Interestingly, those in rural areas with sensory impairments also showed a lower preference for institutional care compared to those without impairments [39]. While informal help initially correlated with greater depressive symptoms, over time, it provided a moderate positive benefit, suggesting that social support, though initially stressful, can become beneficial [32]. Workplace accommodations were common among those with sensory impairments (65.6%), though a notable percentage did not receive or utilize such support (34.4%), emphasizing the variability in access to and use of assistive technologies and employer cooperation [32].

### Income and social protection

Six studies addressed income and social protection in relation to DSL. A population-based study of 5.4 million older adults found that the prevalence of DSL increased as income decreased, ranging from 1.7% among those at 500% or higher of the poverty line to 5.2% among those living below the poverty line [49]. Individuals in lower expenditure quintiles (the lowest expenditure quintile represents the 20% of people who spend the least and may struggle to afford healthcare, assistive devices such as hearing aids and glasses, and other essential services) had higher rates of sensory loss and unmet needs for glasses and hearing aids [30]. One study found that higher income was linked to a lower risk of DSL with social isolation and poor health being significant predictors. Socioeconomic factors explained a substantial variance in DSL prevalence [42]. Another study addressed challenges faced by older adults with combined hearing and vision loss in the workplace. It emphasized the importance of support and resources such as accommodations, assistive technology, higher education levels, and changing jobs or types of work which are associated with continued employment [32]. Notably, individuals with a higher number of adverse childhood experiences had increased odds of experiencing sensory impairments in later life [50].

### Education

Nine studies examined the link between education and DSL which consistently showed that higher education levels were associated with a lower prevalence of DSL. Individuals with college education or higher demonstrated the lowest prevalence of sensory loss. In contrast, those with lower educational attainment, such as primary or secondary schooling, exhibited higher rates of DSL and unmet needs for sensory aids like glasses and hearing aids [46,47,49]. Education also served as a protective factor against depression, a common comorbidity of DSL [25]. Higher education levels correlated with a reduced risk of depression [25]. Furthermore, volunteering was identified as a mitigating factor for depressive symptoms in individuals with DS, highlighting the role of social engagement as a protective factor [51].

Older adults with DSL who had not completed high school diploma reported higher prevalence rates, with many living below the poverty line [25,49]. Social inactivity within educational and community contexts was also prevalent amongst this group, underscoring social challenges faced by individuals with DSL [27]. Additionally, studies revealed that older adults with DSL were more likely to be female, older, illiterate, single, and unemployed. These trends were consistently observed in both urban and rural contexts [39].

### Unemployment and social security

Three studies examined the relationship between unemployment, job security and DSL. Findings revealed that individuals with DSL had significantly higher retirement rates (94.5%) compared to those with no visual impairment and normal hearing (62.5%) [34]. Private income sources were linked to a lower likelihood of DSL, suggesting that financial stability may help mitigate some employment-related challenges faced by this population [41]. Employment was found to reduce depressive symptoms among individuals with DSL, reflecting a trend commonly observed in the general population [39]. However, older adults with DSL often experience higher unemployment rates due to the profound impact of sensory losses on their ability to work, highlighting the substantial barriers to employment faced by this group [32]. Many individuals reported that their sensory impairments required adjustments to their type of work, workload, and even job roles altogether [32]. Overall, the studies underscored the significant burden DSL places on job security and employment opportunities emphasizing the need for targeted interventions and support.

### Other SDH indicators (Indigenous status, food security, race, structural conflict, and working life conditions)

The search did not identify any studies that explored the relationship between Indigenous status, structural conflict, or working life conditions and DSL. However, one study examined the link between food insecurity and DSL. It was found that participants with the poorest diet quality (lowest dietary score quintile) were more than twice as likely to be affected by DSL compared to those with the highest diet quality (top quintile). A clear trend was observed across total dietary score quintiles, with the risk of DSL increasing as dietary quality decreased [52]. This suggests a strong association between lower diet quality and a heightened risk of DSL.

Two studies investigated the connection between race and DSL highlighting disparities in risk of DSL. One study revealed that both White and Black individuals with DSL experienced an increased risk of mobility issues, whereby Black participants were nearly twice as likely to face these difficulties compared to Whites [31]. Among Black participants, visual impairment alone significantly exacerbated mobility challenges. In terms of daily living activities, DSL posed greater barriers for both racial groups, with Black individuals encountering a disproportionately higher risk [53]. Another study found that Hispanic race and ethnicity were associated with elevated rates of visual impairment and DSL, further underscoring the need to address racial disparities in access to sensory care [54].

## Discussion

In this review, we explored key social determinants of health that significantly affect the experiences of older adults with DSL, in relation to the 13 SDHs identified by the WHO and the American, Nordic, Australian, New Zealand, and Canadian governments. Our findings align with the 2023 World Federation of Deafblind Global Report on Older People [55] which highlights the compounding impact of DSL on health outcomes, social participation, and access to essential services. Across all SDH indicators, people with DSL face greater inequities compared to those with only hearing or vision impairment or no disabilities. These disparities heighten vulnerability to poor health outcomes, limit opportunities for education or employment and restrict access to healthcare services and social support networks, further exacerbating isolation and comorbidities. These findings underscore the multifactorial interaction between gender, sensory impairments, and broader social determinants of health, highlighting the need for gender-specific strategies to address the unique challenges faced by people with sensory impairments. These results emphasize the importance of targeted interventions that consider both social and environmental factors, particularly for people with DSL in rural and lower-income settings.

### Education inequities and the need for greater accessibility

Studies have shown that older adults who develop dual sensory loss (DSL) early in life are more likely to have lower levels of education [24,36,51,56–60]. Specifically, 19.5% of individuals had not completed high school, and over one-third (35.9%) of those with DSL lacked a high school diploma [49]. This educational disparity may be due to limited access to educational resources and support during their formative years [55]. Technological and non-technological aids, such as glasses, magnifiers, live assistance, picture boards, electronics, and computer software, are crucial in supporting individuals with DSL in educational settings [55]. Additionally, older adults with higher levels of education tend to have a lower prevalence of sensory difficulties [61].

Suboptimal educational outcomes can have long-term impacts on income, health, and overall quality of life [62]. Therefore, ensuring equal access to disability pensions, social protection programs, assistive devices, and communication supports is vital for improving outcomes for older adults with DSL [55]. However, the reviewed studies did not provide education-specific recommendations, highlighting a significant gap in the literature.

### Social isolation, living alone, and barriers to communication

A noteworthy study focused on the correlation between older adults with dual sensory loss and social factors such as social network diversity, social participation, availability of social support, and loneliness in older Canadians [37]. As people age, they are more likely to experience the loss of a spouse, which can lead to living alone. Living alone can contribute to social isolation and loneliness, which are significant risk factors for various health issues and mortality. The Canadian Longitudinal Study on Aging (CLSA) data supports the notion that older adults living alone may face unique challenges, including higher rates of sensory impairments and associated health risks [63].

According to the World Federation of the Deafblind Global Report 2018 [11], disability may pose barriers to finding romantic partners leading to a higher likelihood of living alone, which in turn increases risk of loneliness and mental health challenges. To address these issues, social programs like befriending initiatives leisure activities and skill development workshops can help foster social interaction and improve mental health [11]. In addition, reduced communication abilities among older adults with DSL negatively impacts their social participation [64]. Communication barriers often arise from a lack of awareness about DSL-specific communication strategies and limited availability of accessible options in both public and private spaces. Community-based interventions promoting social inclusion, the availability of interpreter guides and the use of assistive devices have demonstrated the potential to improve social engagement and communication abilities [11,65]. Collaborative efforts by public and private sectors to enhance the accessibility and affordability of assistive devices could further support this population.

### Co-morbidities and the need for targeted interventions

Older adults with DSL have a significantly higher risk of mortality compared to those without sensory impairments. Additionally, older adults with DSL face an increased risk of depression and anxiety, as highlighted by the World Federation of the Deafblind Global Report 2018 [11]. This risk is closely linked to social isolation and reduced social participation, which are exacerbated by the intersection of disability and age [11,66,67]. Volunteer intervention programs aimed at reducing depressive symptoms could be highly beneficial [51]. Additional longitudinal research is needed to better understand mental health challenges faced by this population and inform improved service provision [18,30,45].

There is a strong association between DSL and cognitive decline [12,36,56,68–70]. It is essential for healthcare providers to be more aware of the increased risks associated with cognitive impairments and DSL. Regular screening, a public health approach that integrates sensory and cognitive assessments, and exploring interventions to prevent or mitigate sensory loss are crucial strategies to reduce these risks. [22,62,70].

### Gaps in research and directions for future studies

While this review provides valuable insights into the associations between SDH and the experiences of older adults with DSL, additional research will address underexplored factors such as working conditions, food insecurity, Indigenous status, race, and structural conflict and age classifications [71]. Understanding the effect of these SDH on the health and quality of life of older adults with DSL is essential for developing a more comprehensive understanding of their lived experiences. The creation of collaborative international government resources offers significant opportunity to analyze tailored data on older adults with DSL. This dataset includes information on disability type and severity, use of aids and assistive devices, unmet needs, accommodations in school or work, and sources of income, providing a rich basis for future research to inform evidence-based interventions that span multiple international contexts. Doing so will work towards prioritizing a unified internationally recognized SDH framework to enable accurate cross-study comparisons. Furthermore, race and Indigeneity are more than demographic variables, acting as proxies for historical and structural inequities such as colonialism, systemic racism, and institutional exclusion that shape access to healthcare, education, income, and social supports as SDHs [72]. For Indigenous populations specifically, DSL may occur in the context of intergenerational trauma, limited culturally appropriate services, and geographic isolation, which compound barriers to care. Structural conflict, including displacement, war, and sociopolitical marginalization, can further exacerbate sensory health disparities, particularly in refugee or post-conflict populations. Ignoring these axes of oppression perpetuates the invisibility of already marginalized groups and fails to generate policy-relevant insights. Future research must adopt an intersectional framework that explicitly analyzes how multiple forms of disadvantage (for example race, gender, disability, poverty, Indigeneity) interact to shape health outcomes. This includes collecting disaggregated data by race and Indigenous identity, involving community members in the research design, and embedding principles of equity, decolonization, and culturally safe care. Employing such intersectional frameworks can be achieved using equity-oriented tools like the PROGRESS framework [73]. Additionally, integrating critical social science perspectives and community-based participatory research methods can illuminate structural determinants that are otherwise missed by traditional epidemiological approaches. Finally, age classification/range differed in most studies included in this paper (S1 File). Future research should adopt standardized age categories for older adults, such as young-old (65–84 years), oldest-old (85–99 years), and centenarians (100 + years) [74]. Studies should provide detailed reporting on the age distribution of participants, including mean age, age range, and age-specific analyses. This will ensure consistency and comparability across studies [75]. From a policy perspective, implications should be context-specific and tailored to unique needs of each setting. For high-income countries, policy-interventions should focus on addressing systemic gaps in care continuity, increasing funding for assistive devices, and expanding inclusive health and social service delivery models. For instance, governments should consider tax incentives or subsidies to employers who accommodate older workers with DSL or mandate accessible housing standards for government-funded senior living and care facilities. In LMICs, policies must prioritize the integration of DSL screening into primary care, invest in low-cost assistive technologies, and employ community-based rehabilitation models that leverage local human resources. Across both settings, these policies must be shaped with people with DSL and their families to ensure culturally safe and disability-inclusive approaches that promote autonomy, dignity, and social participation.

### Limitations

The study’s limitations should be considered when interpreting its findings. First, the search strategy did not identify any articles addressing key SDHs such as working life conditions, structural conflict, and Indigenous status. Consequently, determinants like disability, social-inclusion and non-discrimination were overrepresented while other critical SDHs relevant to older adults with DSL were under explored. In particular, the absence of research on structural conflict and Indigenous status may obscure systemic barriers to education, employment, healthcare services which are known to influence health outcomes in this population. Additionally, race was insufficiently represented, limiting the robustness of the conclusions.

Second, inconsistent age classifications across studies introduced heterogeneity. While this review defined older adults as 60 + , several included studies used a 65 + threshold or included younger participants (n = 18) [20,25,26,29,33,35,37,42,50,52,76–83]. To maintain inclusivity and strengthen synthesis, studies were retained if the sample predominantly comprised older adults (e.g., mean, or median age > 65). Studies without precise age breakdowns were also included when they offered valuable insights into age-related DSL trends.

Finally, the predominance of articles from HICs restrict the generalizability of the findings to LMICs. DSL prevalence varies significantly across regions from 8.59% in North America to 11.03% in Oceania, to 2.32% in Europe and 4.62% in Asia with limited data on Africa [84]. The geographic disparity may mask context-specific determinants such as access to sensory rehabilitation, cultural perceptions of disability, and infrastructural challenges in LMICs. Given the underdeveloped disability support systems in these regions, findings derived from HICs may lack relevance and applicability [85]. Future research should prioritize LMIC context to uncover region specific SDH indicators and promote inclusive methodologies to advance global health equity for older adults with DSL.

### Conclusion

To our knowledge, this scoping review is the first to explore the intersection of SDH and older adults with DSL revealing substantial disparities that heighten vulnerability and restrict access to healthcare, rehabilitation and social participation. The findings underscore the need for further research into unexplored SDH factors using robust, internationally representative data based on standard age-grouping classification and lived experiences of older adults with DSL. Addressing these inequities requires a multifaced approach – by advocacy groups, healthcare providers, researchers and policy makers in enhancing awareness, improving access to services, strengthening social support systems, and promoting inclusive policies – to better support this underserved population and advance health equity.

## Supporting information

S1 Fig PRISMA diagram of studies included in the analysis. (TIFF)

S2 Fig Number of studies published per year. (TIFF)

S1 File PRISMA Scr checklist. (DOCX)

S2 File SDH-DSL supporting data. (PDF)
