# Supplementary figures and images for: Social determinants of health and dual sensory loss in older adults: A scoping review
Source: PLoS One. 2025 Dec 10;20(12):e0338322. doi: 10.1371/journal.pone.0338322 (PMC12694789; doi:10.1371/journal.pone.0338322)

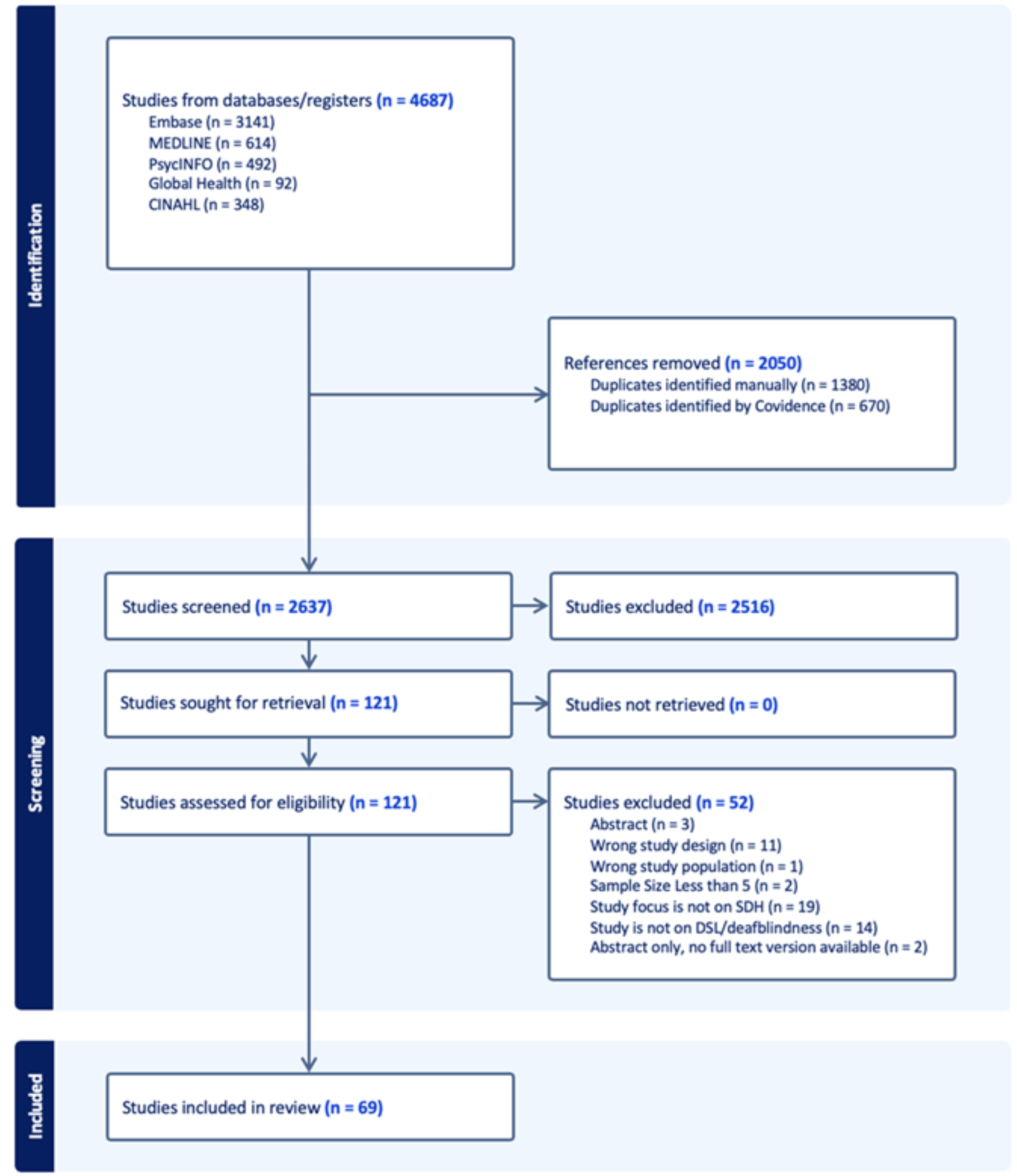

Supplement: S1 Fig — (TIFF) [file pone.0338322.s001.tif]
