# Supplementary material for: Social determinants of health and dual sensory loss in older adults: A scoping review
Source: PLoS One. 2025 Dec 10;20(12):e0338322. doi: 10.1371/journal.pone.0338322 (PMC12694789; doi:10.1371/journal.pone.0338322)
Supplement: S2 File — (PDF) [file pone.0338322.s004.pdf]

TITLE: Social determinants of health and inequities in health for older adults with dual sensory loss worldwide

OBJECTIVE: To understand social determinants of health and how these relate to older adults with dual sensory loss, including living conditions, health,

		ARTICLE DESCRIPTORS								
COVIDENCE NO.	REVIEWER	TITLE OF ARTICLE	AUTHOR, YEAR OF PUBLICATION	LOCATION OF STUDY (COUNTRY)	STUDY POPULATION	METHODS OF DATA COLLECTION	NUMBER OF PARTICIPANTS	AGE GROUP OF PARTICIPANTS	TERM FOR DEAFBLINDNESS	DEFINITION OF DEAFBLINDNESS
Enter the Covidence # provided.	*Enter your name.*	*Enter the name of the article.*	*Format is Last name of the first author, year. For example: Jaiswal, 2021. If there are more than 1 author, add the term "et al." after the first author's last name. For example: Jaiswal et al., 2021.*	*This is the country where the study took place. This can be found in the abstract/methods section of the paper. In this case you will write, [Country name]. Example: Canada If you can't find it, please add the location where the first author is from and add an asterisk. This can be identified through their affiliation. In this case, you will write: [Country name]*. Example: Canada**	*This is the participants that are being studied. For example, this can be older adults with deafblindness. Or individuals aged 5-90 years old with deafblindness. Please indicate the impairments of the study population. For instance, a study with youth with vision, hearing, and DSI. Use the same term that the author of the study used to describe the deafblind population (i.e. DSI, DSL, deafblindness, etc.)*	*This is typically stated in the abstract or methods section. In the article, you want to identify the way the author collected the data. For instance, this can be through interviews, questionnaires, clinical assessments, analyzing a registry. If you are unsure, please write "Unsure."*	*This can typically be found in the abstract/ methods section. Please note, sometimes articles will indicate they recruited XX number of individuals but only XX participated in the study. We are interested in the number of individuals that actually participated in the study. Please indicate the number of participated individuals and include how many of the total participants were individuals with deafblindness. For example: 100 participants, 25 with deafblindness.*	*Specify the age group of deafblindness participants (e.g. 60-80 year olds). If not specified, please write "Not specified."*	*Please indicate if the participants have congenital, acquired, or age-related deafblindness. If there are none, please write "Not specified in study." If you are unsure, please write "Unsure."*	*Please copy and paste how the author defined of the article defined deafblind. If not specified, please write "Not specified."*
956	Ved	Older adults with dual sensory loss in rehabilitation show high functioning and may fare better than those with single sensory loss	Alfaro et al, 2020	Canada	Older adults (61+) attending sensory rehab centres in Montreal with DSL, VL only, or HL only	interRAI Community Health Assessment through self-report, report from caregiver and health professional, and health records	200 participants, 58 had VL only, 69 had HL only, and 73 had DSL	61-85+	Unsure, implied age-related but also talks about congenital, doesn't seem to be specific	Participants were recruited among older adults (61 years old or older) that were attending sensory rehabilitation centres in Montreal and were eligible for sensory rehabilitation services as defined by the Quebec Ministry of Health (i.e., a visual acuity in the better eye with best standard correction of 20/60 (6/18) or less, or a visual field diameter of < 60 degrees in the better eye, or hemianopsia (loss of half of the visual field); and/or an unaided pure-tone average decibel hearing loss (dB HL) in the better ear of 35 dB HL or more across 4 frequencies: 0.2, 1, 2, and 4 kHz .
1143	Ved	"We're really not ready for this": A qualitative exploration of community pharmacy personnel's perspectives on the pharamceutical care of older people with sensory impairment	Alhusein et al, 2019	Scotland	Community pharmacy personnel, asled about interactions with patients having single or dual sensory impairment	Interviews	30 total,17 community pharmacists and 13 other	NA	Not specified in study	NA
1326	Ved	"Has she seen me?": a multiple methods study of the pharmaceutical care needs of older people with sensory impairment in Scotland	Alhusein, 2018	Scotland	23 older people, 13 with dual impairment, 5 of each hearing or vision loss, and 30 community pharmacy personnel with 171 total survey responses	Interview and survey	23 older people, 13 with dual impairment, 5 of each hearing or vision loss, and 30 community pharmacy personnel with 171 total survey responses	67-95 years	Not specified in study	NA
794	Ved	Hearing and vision care provided to older people residing in care homes: a cross-sectional survery of care home staff	Andruskjak, 2021	England	Care home staff	Cross-sectional survey, sampled via email and paper	400 staff total, 46% said they used screening tools to identify hearing and vision impairments (43.8%).	NA	Not specified in study	NA
498	Ved	Associations of Dual Sensory Impairment with Incident Mobility and ADL Difficulty	Armstrong et al., 2022	USA	Older adults aged 70-79	COX proportional hazard models through participant interview, hospital records, or proxy interviews	2020, 23% had DSI but 25 had incident mobility difficulty and 224 had incident ADL difficulty	70-79	Not specified in study	Vision impairment (VI) was defined as impaired visual acuity (20/50 or worse on Bailey-Lovie distance test) and contrast sensitivity (25 decibels. M

	SOCIAL DETERMINANTS OF HEALTH (EXTRACT FROM RESULTS SECTION OF PAPER)								
COVIDENCE NO.	INCOME AND SOCIAL PROTECTION	EDUCATION	UNEMPLOYMENT & JOB SECURITY	WORKING LIFE CONDITIONS	FOOD INSECURITY	HOUSING, BASIC AMENITIES, AND THE ENVIRONMENT	SOCIAL INCLUSION AND NON-DISCRIMINATION	STRUCTURAL CONFLICT	ACCESS TO AFFORDABLE HEALTH SERVICES OF DECENT EQUALITY
Enter the Covidence # provided.	*Copy and paste the section of the results section of the article that examines/include aspects of income and social protection in individuals with DSL. Income can impact health but also the importance of income as a means of gaining access to other social determinants of health such as education, food, housing, recreational activities, and other societal resources. Social protection covers the range of policies and programmes needed to reduce the lifelong consequences of poverty and exclusion. This includes grants or vouchers to connect families/ individuals with health care, nutritious food and quality education. If not discussed, write NA.*	*Copy and paste the section of the results section of the article that examines education in individuals with DSL. Education is a recognised social determinant of health and directly influences the other factors later in life that contribute to health such as being able to gain solid employment, accessing stable housing and being able to make healthy food choices. If not discussed, write NA.*	*Copy and paste the section of the results section of the article that examines unemployment and job security in individuals with DSL. Unemployment is when people are jobless, actively seeking work, and available to take a job. Job security is having a job that is secure and from which one is unlikely to be dismissed. If not discussed, write NA.*	*Copy and paste the section of the results section of the article that examines working life conditions. Working life conditions refers to the working environment and all existing circumstances affecting labor in the workplace, including job hours, physical aspects, legal rights and responsibilities. If not discussed, write NA.*	*Copy and paste the section of the results section of the article that examines food insecurity in individuals with DSL. Food insecurity is described as the inability to acquire or consume an adequate diet quality or sufficient quantity of food in socially acceptable ways, or the uncertainty that one will be able to do so. Household food insecurity is often linked with the household's financial ability to access adequate food. If not discussed, write NA.*	*Copy and paste the section of the results section of the article that examines housing, basic amenities, and the environment. Housing as a social determinant of health looks at adequate housing conditions, affordability, and residential stability. Basic amenities include clean drinking water, food, electricity, etc. If not discussed, write NA.*	*Copy and paste the section of the results section of the article that includes social inclusion/non discrimination in individuals with DSL. Social inclusion is defined as the process of improving the terms of participation in society, particularly for people who are disadvantaged, through enhancing opportunities, access to resources, voice and respect for rights. Non-discrimination is ensuring fairness in treating people without prejudice. If not discussed, write NA.*	*Copy and paste the section of the results section of the article that examines structural conflict in individuals with DSL. Structural Conflict is described as when there is the loss of human rights and breaches of medical neutrality as a result of conflict or emergency responses. If not discussed, write NA.*	*Copy and paste the section of the results section of the article that examines access to affordable health services of decent equality in individuals with DSL. This is described as when every person who shares the same type and degree of health need must be given an equally effective chance of receiving appropriate treatment of equal quality so long as that treatment is available to anyone. If not discussed, write NA.*
956	DSL diminishes a person's capacity to communicate, acquire information, perform daily activities, and fully participate in social environments without assistance or support (e.g., sign-language interpreter) [3–5]. Persons with DSL are at higher risk for numerous health issues including cognitive impairment, social isolation, impaired mobility, and depression [2,3,6]. Furthermore, persons with DSL are more likely to be unemployed, have low socio-economic status and poorer educational outcomes, compared to persons with other disabilities	NA	NA	NA	NA	NA	Most participants in all groups (82.8% to 87.7%) did not trigger the Social Relationships CAP (Table 5), indicating that they were not at risk of reduced social relationships and did not require intervention to facilitate social engagement, χ2 (2, N = 200) = 0.73, p = 0.82. In the items regarding psycho-social well-being (Table 4), most participants in all sensory impairment groups were not experiencing issues such as loneliness and neglect. Only the item"conflict or anger with family or friends" was associated with sensory impairment group (Fisher's p = 0.04). However, this association was no longer significant after controlling for age (χ2(10, N = 200) = 16.15, p = 0.09), cognition (χ2 (10, N = 200) = 17.53, p = 0.06), and risk of depression (χ2 (10, N = 200) = 14.58, p = 0.14)<br><br>Most participants (91.4% to 98.6%; Fisher's p = 0.54) were independent in completing their ADLs (Table 4). [illegible]	NA	NA
1143	NA	Na	NA	NA	NA	NA	NA	NA	This study revealed that most interviewees addressed either hearing or sight impairment; there was little mention of dual sensory impairment. This points to the need for improving guidelines and resources for community pharmacy personnel to help people with a dual sensory impairment.
1326	NA	NA	NA	NA	NA	NA	NA	NA	Changing appearance of medicines, lack of training, lack of contact, patients masking senosry loss, difficulty communicating, lack of time, pt not understanding pharmacy, lack of available staff, patients not understanding medicines, lack of availability of assistive technology, accessibility of pharmacy environment are barriers for accessing pharmaceutical care across all stages of treatment journey leading to suboptimal care
794	NA	NA	NA	NA	NA	NA	NA	NA	Our study results showed that whilst some practices known to support residents hearing and vision [4] are commonly adopted by care homes, others were reported as being rarely found to be used. Practices commonly reported to be used include; recording sensory loss in residents' care plans, annual professional vision tests, regularly checking hearing aids and regularly checking glasses. The majority of respondents also reported having quiet areas and well-lit rooms in their care home. These responses show that some practices suggested by existing guidelines are being adhered to [19]. However, there are certain practices which are known to assist with residents' ear and eye care that were reportedly rarely used, including use of screening tools for both hearing and vision, hearing assessments by audiologists, and access to other assistive devices. This reveals that interventions aimed at improving practice, including use of [illegible]
498	NA	NA	NA	NA	NA	NA	NA	NA	NA

463	Ved	Associations of dual sensory impairment with long-term depressive and axienty symptoms in the United States	Armstrong et al., 2022	USA	Come from HABC Study	Multinominal logistic regression models and for depressive symptoms, used ≥8 on the 10-item Center for Epidemiologic Studies-Depression Scale and for anxiety Hopkins Symptom Checklist. Self-reported	2102, 51.9% female, 538 had no vision or hearing impairment, 743 had hearing impairment only, 330 had vision impairment only, and 491 had dual sensory impairment	74 (SD=2.8 years)	Old-age, but asked if caused by conditions like diabetic retinopathy or cataracts or glaucoma	audiometric findings were used to define hearing status at study baseline (Year 1). Using headphones at frequencies from 0.25 to 8 k Hertz (kHz) (TDH 39;Telephonics Corporation) and an audiometer (MA40; Maico Diagnostics) calibrated to the standards of the American National Standards Institute (S3.6- 1996), pure-tone air conduction thresholds were collected in both left and right ears. A four-frequency pure tone average (PTA) was calculated from audiometric threshold at 0.5, 1, 2, and 4 kHz for the better hearing ear. HI is defined as PTA > 25 dB, since this cut-off has been used in previous Health ABC studies. The Bailer-Lovie distance visual acuity test, converted to Snellen equivalents was used, with an acuity equivalent of 20/50 or worse being defined as impaired. Additionally, Pelli-Robson contrast sensitivity was used, with 1.3 log units or less being defined as impaired (log Contrast units, higher values = better sensitivity) . VI was defined as having impaired visual acuity or contrast sensitivity. Dual Sensory Impairment. Using these defined cut-offs for HI and VI, we created a categorical variable to examine DSI: both hearing and vision, hearing only, vision only, and neither (reference). Both hearing and vision are time-invariant, as they were measured once in HABC.	NA	NA	NA	NA	NA	NA	Most of the sample (57.7 %; n = 1212) did not have any components of low social support (dissatisfaction, low emotional support, and low social contact). About 18.1 % of the sample reported low emotional support only, and 15.4 % reported low social contact only. All other combinations and dissatisfaction only were all below 5 %. In Table 1, approximately 1.1 % (n = 23) had all three components of low social support; 6.9 % (n = 145) had two components of low social support; 34.4 % (n = 721) had one component of low social support. The frequencies did not differ between Years 1 and 5. There were no associations between the DSI categories and number of components of low perceived social support cross-sectionally in Years 1 and 5, except for VI and one component of low perceived social support in Year 1 (OR = 1.41, 95 % CI: 1.03, 1.92)	NA	NA
715	Ved	Self-reported dual sensory impairment, dementia, and functional limitation in Medicare beneficiaries	Assi et al, 2021	USA	Adults 65+ with single sensory impairment, no sensory impairment, or dual sensory impairment	Cross sectional analysis of National Health and Aging Trends Study from 2015 and survey weighted Poisson regression models	7124, 43.9% were 75+ and 55.3% were female, 4843 had no sensory impairment, 1988 had single sensory impairment, and 293 had both	NA	Age-related and included acquired since they included AD patients	Vision impairment was defined as self-reported blindness, or not being able to see across the street and/or newspaper print (despite using glasses or contacts if applicable). Hearing impairment was defined as any of the following: self-reported deafness, using a hearing aid or other hearing device, or not being able to hear well enough to use the telephone or to carry on a conversation in a room with a radio or TV playing (using a hearing device if applicable). Dual sensory impairment status was categorized as having no sensory impairment (reference), a single sensory impairment (i.e., vision or hearing impairment), or dual sensory impairment.	NA	NA	NA	NA	NA	NA	NA	NA	NA
898	Ved	Assessment of Sensory Impairment and Health Care Satisfaction Among Medicare Beneficiaries	Assi et al, 2020	USA	Medicare beneficiaries, those categorized as no sensory impairment, hearing impairment, vision impairment, dual sensory impairment	Cross sectional study used data from 2017 Medicare Current Beneficiary Survey and it was self-reported functional sensory impairment	10783, 4250 had no impairment, 2822 had HI only, 1557 had VI only, and 2154 had DSI	65-75+	Self-reported so age-related	Participants were asked to describe their vision and hearing by selecting 1 of the statements: "no trouble," "a little trouble," or "a lot of trouble" seeing and/or hearing. Functional vision impairment was defined as any self-reported trouble seeing ("a little trouble" or "a lot of trouble") with the use of glasses or contact lenses when applicable. Functional hearing impairment was defined as any self-reported trouble hearing with the use of hearing aids when applicable.	an income poverty ratio between 120% and 135% or equal to or greater than 200% (vs 100% of the FPL: OR, 1.12; 95% CI, 0.73-1.72; P = .06) was not associated with lower odds of dissatisfaction with quality of care	NA	NA	NA	NA	NA	NA	NA	In the adjusted model, those with dual sensory impairment had higher odds of dissatisfaction with quality of care relative to no sensory impairment (OR, 1.52; 95% CI, 1.10-2.10; P = .01); the odds were were not significantly higher for those with hearing impairment (OR, 1.31; 95% CI, 0.88-1.95; P = .19) and vision impairment only (OR, 1.17; 95% CI, 0.79-1.74; P = .43). In fully adjusted models, those with dual sensory impairment had the highest odds of dissatisfaction compared with those without sensory impairment across outcomes. greatest odds of dissatisfaction among those with dual sensory impairment were for dissatisfaction with doctors' concern with overall health (OR, 2.03; 95% CI, 1.55-2.66; P < .001). Those with hearing impairment only also had greater odds of dissatisfaction with their doctors' concern (OR, 1.38; 95% [illegible]
1102	Ved	Decline in Instrumental Activities of Daily Living Over 4-Year: The association with hearing, visual, and dual sensory impairments among non-institutionalized women	Bouscaren et al, 2019	France	French E3N-elderly subcohort , hearing and visual impairments	Self-reported measurements of sensory imapirment in 2006 and 2010	4010 community dwelling older women, 3903 had no visual impairment, 107 had visual impairment, 1919 had no hearing impairment, 2091 had hearing impairment	Women born between 1925 to 1930	Age-related and included acquired since they included AD patients	Visual impairment was defined as self-reported difficulties in reading a newspaper and/or recognizing a 4-metre far person, with correcting devices if needed. . Hearing impairment was assessed through self-reported difficulties in listening to and understanding a conversation in a noisy environment, with correcting devices if needed. To operationalize dual sensory impairment and explore the effect associated with	NA	NA	NA	NA	NA	NA	NA	NA	Among the 2,091 women who reported a hearing impairment, 1,535 (73.4%) had no hearing aids, and 556 (24.6%) had hearing aids that insufficiently corrected the impairment. All women reporting visual impairments (n=107) had visual devices that insufficiently
264	Ved	Dual Sensory Impairment and Cognitive Impairment in the Korean Longitudinal Elderly Cohort	Byeon et al, 2021	South Korea	Prospective community-based elderly cohort (Korean Longitudinal Study on Cognitive Aging and Dementia)	Self-report questionnaire	6520 total, 932 had normal sensory function, 2957 had single impairment, 2631 had DSI	58-101	In the context of dementia and normal aging but also accounted for prsecnce or absecnce of eye and ear disease	Participants were asked to self-evaluate their hearing. "Normal" was 0, "Reduced but able to communicate without using a hearing aid" was 1,"Reduced so that communication is possible only using hearing aids" was 2, "Difficulty communicating even when hearing aid is used" was coded as 3, and "I can't hear at all" was coded as 4. In the case of visual function, "Normal" was 0, "Reduced, but able to view newspaper or television without wearing glasses or lenses" was 1, "Reduced, so that I can view newspaper or television only with glasses or lenses" was 2, "Reduced. As a result, I can't view newspaper or television even if I wear glasses or lenses" was 3, and "I can't see at all" was 4. In this study, 0 was defined as "normal" and 1, 2, 3, and 4 were defined as "sensory impairment."	NA	NA	NA	NA	NA	NA	NA	NA	NA
2794	Ved	Association of sensory impairment with cognitive function and mental health among the older adults in china	Chen & Zhou, 2023	China	Participants of 2018 Chinese Longitudinal Healthy Longevity Survey based on self-reported hearing and vision function	Self-report questionnair from CLHLS and face to face interviews by trained enumerators or doctors/nurses and if unable to answer, was collected from spouse or close fmaily member	11722 total, 19.2% was HI, 14.6% was VI, and 15.3% was DSI	65+	Not specified in study	For HI, we selected a short questionnaire that inquired the participants "Do you have any difculty with your hearing?". The possible responses were "I have hearing difculty" and "I do not have any difculty with my hearing". For VI, we selected an item that asked the participants "Can you see the circle on the card and distinguish the direction of the break in the circle with a fashlight shining on it." The participants were categorized as having vision impairment if they could not distinguish, could not see, or were blind.	NA	NA	NA	NA	NA	NA	NA	NA	NA

1368	Ved	The association amongst visual, hearing, and dual sensory loss with depressoin and anxiety over 6 years: The Tromso Study	Cosh et al, 2018	Norway	Participants of longitduinal population based Tromso study	Survey, attendence at interview, and medical examination	Total 2156, 543 had sensory loss for vision, 285 had hearing loss, 6.8% had DSL	60+, mean was 66.9 SD=5.2 years	Not specified in study	Visual acuity was assessed using Snellen charts at a distance of 6 m. Assessments were undertaken using participants' usual optical correction, where applicable. A Snellen score of	NA	NA	NA	NA	NA	NA	NA	NA	NA
3131	Ved	Communication participation in older adults with dual sensory loss	Crowe et al, 2020	Denmark	Older adults	Questionnaire, used oral communication	Total 513, 62% had residual functional vision, 80% had residual functional hearing	50-102, mean of (82 SD=12.6) years, 74% female	Not specified	Simply said Nordic definition	NA	NA	NA	NA	NA	NA	In terms of communication participation, nearly all participants using oral communication reported being able to engage in one-to-one conversation (n = 265, 97%) and the majority could participate in a group conversation (n = 165, 60%. The majority of oral communicators also reported that they withdrew from conversation (n = 188, 70%) and were alone when they would prefer to be with other people (n = 194, 71%). Over half of the participants did not participate in social activities outside the home once a week or more (n = 121, 44%). An association was found between severity of hearing loss and two communication participation variables, with participants with mild or moderate hearing loss significantly more likely to be able to participate in group conversations [illegible]	NA	NA
1298	Ved	Older Adults with a Combinatoin of Vision and Hearing Impairment Experience Higher Rates of Cognitive Impairment, Functional Dependence, and Worse Outcomes Across a Set of Quality Indicators	Davidson, 2019	Canada	Older home care clients	Resident Assessment Instrument for Home Care	Total 352,656, 72188 had DSI	65+, mean was 82.8 SD=7.9 years	Older age, in the context of Alzheimer's and Dementia	The hearing and vision items can be combined to create the Deafblind Severity Index (DbSI), which is used to identify DSI. The DbSI creates a score from zero (no impairment on either sense) to five (severe impairment on both senses), to determine the level of DSI (Dalby et al., 2009). Clients were classified as having DSI (score of 3+) if they experienced at least a mild impairment on both of the hearing and vision items. The individual hearing and vision items used in the DbSI have excellent test–retest reliability (hearing: kappa = 0.83; vision: kappa = 0.85) (Dalby et al., 2009). Previous research has shown preliminary evidence of concurrent validity, as a higher score of the DbSI coincides with greater difficulties with IADLs and interacting with others (Dalby et al., 2009). Clients that were not considered to have DSI (DbSI score of	NA	NA	NA	NA	NA	NA	In terms of communication ability, home care clients with DSI were 2.5 times more likely to be associated with moderate/severe difficulty understanding others (2.49; CI = [2.45, 2.53]) and over twice as likely to be associated with moderate/severe difficulty being understood by others (2.12; CI = [2.08,2.15]) (Table 1). They were also 72% (1.72; CI = [1.70, 1.75]) more likely tobe associated with a decline in their communication ability in the last 90 days and loneliness (1.29; CI = [1.26, 1.31]) compared with clients without DSI. . Clients with the greatest degree of sensory impairment (i.e., highly impaired hearing and severely impaired vision) had the overall highest rates of impaired ADLs (78.2%) and moderate/major difficulty performing IADLs (95.0%). The rates incrementally increased as the client became more impaired in their hearing/vision, with the highest prevalence always occurring with highly impaired hearing	NA	NA
1532	Ved	Psychosociual well-being and health-related qauality of life in a UK population with Usher syndrome	Dean et al, 2017	United Kingdom	UK residents with Usher syndrome	Online survey distributed through deafblind-related charities, support groups, and social groups	90, 8.9% were 66+ and 13.3% were 56-65 years old	18+	Usher Syndrome	Level of hearing loss was assessed by asking participants which of the following (mild, moderate, severe/profound, unknown) best described their level of deafness. Categories used are those used within UK healthcare to define levels of deafness.38 Participants were also asked to identify their sight registration status (partially sighted, blind/severely sight impaired, unknown). This was based on UK categories for the registration of impaired sight.39 Finally participants were asked a single self-rated question about whether they had any other disabilities and health illnesses (yes or no)	NA	NA	NA	NA	NA	NA	. Those variables that were most significantly associated with mental HRQOL were depressive symptoms, loneliness and social support (see online supplementary appendix I). Depressive symptoms (β=−0.60, p<0.001) and loneliness (β=−0.20, p<0.05) were associated with poorer HRQOL. Whereas social support was associated with better mental HRQOL (β=0.19, p<0.05). Previous research has suggested that the poor psychological well-being of people with dual sensory impairment is due mostly to their experience of social isolation.49 Our results indicate that as levels of loneliness increase that the mental HRQOL of our population decreased.	NA	NA
1176	Ved	Association of Sensory and Cognitive Impairment with Healthcare Utilization and Cost in Older Adults	Deardorff et al, 2019	USA	Community-dwelling Medicare beneficiaries	Self-report survey from Medicare Current Beneficiary 1999-2006	24009 total , 5854 had hearing impairment, 3488 had vision impairment, 4375 had DSI	65+	Dementia or normal aging	HI was defined based on two self-reported questions: (1) Which statement best describes your hearing (with a hearing aid, if you use one)? (no trouble, a little trouble, or a lot of trouble) and (2) Do you use a hearing aid (yes, no, or deaf)? If participants reported “a little trouble” or “a lot of trouble” or if they used hearing aids or indicated deafness, then the participants were classified as hearing impaired. VI was defined based on one self-reported question: How much trouble do you have with your vision? (no trouble, little trouble, or a lot of trouble). Subjects who reported “little trouble” or “a lot of trouble” were classified as visually impaired. We did not define VI based on reported use of glasses or contacts because 83% of subjects reported using glasses, suggesting that many respondents referred to reading glasses.	NA	NA	NA	NA	NA	NA	NA	NA	Among participants with dementia, no statistically significant differences in spending were seen in the fully adjusted model among the different combinations of sensory impairment. Among those without dementia, small, but statistically significant, differences in total spending were generally seen in participants with all combinations of sensory impairment compared to those without any sensory impairment. In the fully adjusted model, participants with neither sensory impairment nor dementia spent an average of $1056 per year compared to $1099 among those with VI alone (P = .063), $1120 among those with HI alone (P = .002), and $1151 among those with DSI (P < .001). Results for average annual Medicare FFS cost per participant in 2006 dollars are displayed in Table 4. Those with dementia had higher annual Medicare FFS cost compared [illegible]

28	Ved	The association between dual sensory loss and healthcare expenditure: Mediating effect of depression	Du et al, 2024	China	Participants of survey	2018 CHARLS Database (China Health and Retirement Longitudinal Study)	13412, 58.96% had DSL, 13.96% had no problem	45+	Self-reported so age-related	Within the CHARLS questionnaire, respondents were asked to rate their eyesight for both near and distance vision on a 5-point scale ranging from “excellent” to “poor”. Participants who responded with “fair” or “poor” for either question were categorized as having vision loss. The question related to hearing condition is: “Is your hearing very good, good, fair, poor, or very poor (with a hearing aid if you normally use it and without if you normally don't)?” A “fair” or “poor” answer is also classified as hearing loss. DSL is defined as the presence of both vision and hearing loss	NA	Age and education are protective factors for the development of depression. People aged 60–69 (OR = 0.886, 95%CI:0.818–0.965) and 70–110 years old (OR = 0.818, 95%CI:0.743–0.900) are less likely to have depression compared to people aged 45–59 years old. With higher education, people would have a less risk of depression, those who are educated in primary school (OR = 0.830, 95%CI:0.756–0.912), middle school (OR = 0.861, 95%CI:0.781–0.949) and high school and above (OR = 0.657, 95%CI:0.580–0.745) are less likely to feel depressed. Table 4 shows the influence of DSL on participant outpatient OOP, inpatient OOP and CHE and the corresponding mediating effect of depression.	NA	NA	NA	NA	NA	NA	In model 1, the presence of DSL is associated with higher outpatientOOP costs and inpatient-OOP costs as well as a higher incidence of CHE (β for outpatient-OOP cost = 2366.87, 95%CI: 1752.16–2981.58; β for inpatient-OOP cost = 1032.71, 95%CI: 893.67–747.74; OR for CHE = 1.805, 95%CI: 1.555–2.095; all P < 0.05). When depression was included in modle2, DSL is still associated with increased healthcare costs and CHE (β for outpatient-OOP cost = 1922.35, 95% CI:1303.38–2541.32; β for inpatient-OOP cost = 1041.04, 95%CI: 610.72–1471.35; OR for CHE = 1.655, 95%CI: 1.424–1.924; all P < 0.05), and depression also associated with higher healthcare costs and higher incidence of CHE (β for outpatient-OOP cost = 2001.19, 95%CI: 1626.28–2376.11; β for inpatient-OOP cost = 1217.05, 95%CI = 948.18–1485.91; OR for CHE = 1.471, [illegible]
2006	Ved	Impairments in hearing and visoin impact on mortality in older people: the AGES-Reykjavik Study	Fisher et al, 2014	Iceland	Participants of AGES-RS, population-based study design	Vision and hearing exams were completed	Total 4926, 25.4% had HI, 9.2% had VI only, 7% had DSI	66-96	Not specified	Not specified	NA	NA	NA	NA	NA	NA	NA	NA	NA
480	Ved	The Association Between Hearing Impairment, Vision Impairment, Dual Sensory Impairment, and Serious Cognitive Impairment: Findings from a Population-Based Study of 5.4 Million Older Adults	Fuller-Thomson et al, 2022	USA	Merged data from 10 consecutives from 2008 to 2017 of American Community Service	Merged data from 10 consecutives from 2008 to 2017 of American Community Service	Total 5405135, 4360981 had no sensory impairment, 666275 had hearing impairment, 198998 had vision impairment, 178881 had both hearing and vision impairment	65+	Not specified	Hearing impairment was based upon the question, “Is this person deaf or does he/she have serious difficulty hearing?” (yes/no). Vision impairment was based upon the question, “Is this person blind or does he/she have serious difficulty seeing even when wearing glasses?” (yes/no). The questions were based on self-report for those who were able to answer, but among those were incapable of answering, a proxy such as a family member or nursing home staff member provided the information.	When those with cognitive impairment were compared to those without cognitive impairment, the prevalence of hearing impairment only (22.2% versus 10.8%), vision impairment only (9.9% versus 3.1%), and dual sensory impairment (16.0% versus 1.9%) was much higher, and the prevalence of those without any sensory impairment was much lower (51.9% versus 84.3%; p < .001). The prevalence of dual sensory impairment increased from 1.5% among those aged 65 to 74, to 2.6% among those aged 75–84, to 10.8% among those aged 85 and older. There was a dose response relationship between poverty level and dual sensory loss, ranging from 1.7% of those at 500% or higher of the poverty line to 5.2% of those living below the poverty line. Older adults who had not completed high school had a higher prevalence of dual sensory impairment than high school graduates or university graduates (6.3%, 3.1%, 1.8%, respectively). Those with missing data on income, many of whom may have been in nursing homes where their information was provided by proxies such as nursing staff, had particularly high levels of dual sensory impairment (13.3%)	Overall, 19.5% had not completed high school, but more than one third (35.9%) of those with dual sensory impairment were without a high school diploma. In the total sample, 9.1% were below the poverty line. This increased to 13.7% in those with dual sensory impairment	NA	NA	NA	NA	NA	NA	NA
775	Ved	Longitudinal Association Between Hearing Loss, Vision Loss, Dual Sensory Loss, and Cognitive Decline	Ge et al, 2021	USA	Merged data from Health nad Retirment Study and Aging, Demographics, and Memory Study	Survey, HRS TICS administered by phone or face to face interview	Total 295, 40.7% had hearing loss, 38.98% had vision loss, 17.63% had dual sensory loss	73-100, mean of 81.5 (SD=5.56 years)	Unspecified	Hearing loss was measured in ADAMS by administering the pure tone thresholds test. The pure tone stimuli were presented at 0.5, 1, 2, and 4 kHz at both 25 and 40 dB to the right and the left ear individually. If a participant reported being able to hear the 25 or 40 dB stimulus at a specific Hz level, then the response was documented “Yes” for that frequency and decibel level, otherwise was “No.” The thresholds for hearing loss severity were defined based on the American Speech-Language Hearing Association guidelines and previous literature.8,30 Based on these criteria, we dichotomized hearing status as: (1) having hearing loss if the better ear could not hear 25 dB at any frequency or (2) having normal hearing if the better ear could hear 25 dB at all or some frequencies. Vision loss was measured in ADAMS by using a Snellen chart. Vision loss was defined as having corrected binocular vision worse than 20/40, normal vision was defined as having corrected binocular vision better than or equal to 20/40. This cut-point has been widely used in other studies of vision loss, including large-scale population-based surveys.This cut-point has also been used in screening for issuing unrestricted driver's licenses. Dual sensory loss was defined as having both hearing and vision loss.	NA	NA	NA	NA	NA	NA	NA	NA	NA
1546	Ved	Visual and hearing imapirment and retirement in older adults: A population-based cohort study	Gopinath et al 2017	Australia	Participants of Blue Mountains Eye Study	Face to face interview with trained personnel, comprehensive data including medical history, demographic, SES, and lifestyle/behaivours.	Total 2409 participants, 32.4% had hearing loss, 23.7% had visual impairment	Average age of 67.2 (SD=8.9%)	Old age	Pure-tone audiometry was performed by audiologists in soundtreated booths, using TDH-39 earphones and Madsen OB822 audiometers (Madsen Electronics, Denmark). Bilateral hearing impairment was determined as the pure-tone average of audiometric hearing thresholds at 500, 1000, 2000, and 4000 Hz (PTA0.5–4kHz) in the better ear, defining any hearing loss as PTA0.5–4kHz > 25 dB HL; mild hearing loss as PTA0.5–4kHz > 25–40 dB HL; and moderate to severe hearing loss as PTA0.5–4kHz > 40 dB HL. An audiologist asked questions about history of any self-perceived hearing problem, and if a hearing aid had been provided. Specifically, participants were asked: ‘Do you or have you ever worn a hearing aid?’ (Yes/No/Don't know). The Hearing Handicap Inventory for the ElderlyShortened version (HHIE-S), developed by Ventry and Weinstein [21], was also administered. The HHIE-S includes 10 questions and a response of ‘yes’ is given 4 points; ‘sometimes’ is given 2 points, and ‘no’ is given 0 points. Scores ≥8 were taken to indicate the presence of a hearing handicap. Monocular distance logMAR (logarithm of the minimum angle of resolution) visual acuity was measured with forced-choice procedures using a retroilluminated chart with automatic calibration to 85 cd/m2 (Vectorvision CSV-100™; Vectorvision Inc, Dayton, OH) according to the Early Treatment Diabetic Retinopathy Study protocol [20]. This was conducted with habitual correction (presenting visual acuity, with current eyeglasses, if worn) and after subjective refraction (bestcorrected visual acuity). For each eye, visual acuity was recorded as the number of letters read correctly from 0 to 70. For the present study, any visual impairment was defined as presenting or best-corrected visual acuity of the better eye less than 39 letters (< 20/40). DSI was [illegible]	NA	NA	Participants with both presenting visual impairment and any level of hearing loss had the highest retirement rate i.e. 94.5% (n = 257) compared to those with no presenting visual impairment and normal hearing function – 62.5% (n = 830). Participants with no presenting visual impairment but who had moderate to severe hearing loss had greater odds of reporting retirement compared to those with no visual and hearing impairment (Table 3), multivariable-adjusted OR 2.52 (95% CI 1.13–5.64). Similarly, participants with no best-corrected visual impairment but with moderate to severe hearing loss had increased likelihood of reporting retirement compared to those with no sensory loss (Table 4), multivariable-adjusted OR 2.15 (95% CI 1.10–4.21). No other significant associations were observed between best-corrected visual impairment and/or hearing loss and retirement (Table 4). Analysis of covariance (ANCOVA) showed that [illegible]	NA	NA	NA	NA	NA	NA
1965	Pineal	Association between diet quality with concurrent vision and hearing impairment in older adults	Gopinath et al., 2014	Australia	Older adults aged ≥50 years with dual sensory impairment (DSI), defined as concurrent vision and hearing impairment	*Semi-quantitative food frequency questionnaire*	2,443 participants. Number with DSI not specifi	≥50	Age-related deafblindness	Visual impairment was defined as visual acuity less than 20/40 (better eye), and hearing impairment as average puretone air conduction threshold greater than 25 dB hl (500-4000 hz, better ear).	NA	NA	NA	NA	Participants in the lowest quintile compared to those in the highest quintile (reference group) had a significantly higher likelihood of having dSi, multivariable adjusted or 2.62 (95% ci 1.08-6.36). additionally, a significant trend was observed with increasing tdS i.e. from the first to the fifth quintile (p-trend = 0.04).	NA	NA	NA	NA
2974	Pineal	Self-Rated Health, Cognition, and Dual Sensory Impairment Are Important Predictors of Depression Among Home Care Clients in Ontario	Guthrie et al., 2015	Canada	Older home care clients (aged 65+) with DSI	Resident Assessment Instrument for Home Care (RAI-HC)	218,850 participants. Number with DSI not spe	65+	Age-related deafblindness	Not specified	NA	NA	NA	NA	NA	NA	NA	NA	NA

1688	Pineal	The Health and Well-Being of Older Adults with Dual Sensory Impairment (DSI) in Four Countries	Guthrie et al., 2016	MULTI: Canada, US, Finland, Belgium	DSI among older adults (65+)	interRAI Assesment	80,634. All participants have DSL.	65+	Age-related deafblindness	Not specified	NA	NA	NA	NA	NA	NA	NA	NA	NA
926	Pineal	Dual sensory impairment and psychosocial factors. Findings based on a nationally representative sample	Hajek et al., 2020	Germany	Middle-aged and older adults with dual sensory impairment (DSI), defined as the combined presence of hearing and visual problems.	Data were used from the sixth wave of the nationally representative German Ageing Survey (DEAS). The study employed widely established scales for quantifying psychosocial correlates and sensory impairments.	5,081 participants with 802 DSI.	40 to 98 years old.	Age-related deafblindness	Not specified	NA	NA	NA	NA	NA	NA	NA	NA	NA
1011	Pineal	The impact of sensory imp	Harithasan et al., 2020	Malaysia	Community-dwelling older adults aged 60 years or older, including those with hearing loss only, vision loss only, and dual sensory impairment (DSI).	Data were collected through assessments including the Geriatric Depression Scale, Revised UCLA Loneliness Scale, Satisfaction with Life Scale, and Mini-Mental State Examination.	229 participants with 19 DSI.	60 years and older.	Age-related deafblindness	Not specified	NA	NA	NA	NA	NA	NA	Nonsensory factors contributed significantly to depression and loneliness scores.	NA	NA
259	Pineal	Older Women in Australia: Facing the Challenges of Dual Sensory Loss	Heine et al., 2020	Australia	Older women aged 65 years and over, experiencing dual sensory loss.	Data were derived from the Melbourne Longitudinal Studies on Healthy Ageing Program, which included assessments via structured questionnaires on health, depression, perceived social activities, community service use, and ageing in place.	1,000 participants at the start of the study in 19	65 years and older.	Age-related deafblindness	DSI was defined based on self-reported impairments in hearing and vision.	NA	NA	those with private income sources were less likely to have DSL	NA	NA	those living alone were more likely to have DSL	poor hearing and DSL, but not poor vision, had a significant influence on perceived inadequate social activities indicating that social participation and independence can be improved for those with poor hearing and DSL.	NA	NA
2575	Pineal	Sensory Loss in China: Prevalence, Use of Aids, and Impacts on Social Participation	Heine et al., 2019	China	Older adults aged 60 years and over from the Chinese population, focusing on those with vision and hearing impairments.	Data were collected using the China Health and Retirement Longitudinal Study (CHARLS), a population-based survey that included measures of vision and hearing loss, use of aids, and social participation.	8268 participants with 4298 DSI.	60 years and older.	Age-related deafblindness	DSL is defined as "the acquired loss, in various degrees of severity, of both vision and hearing acuity, associated with aging and prevalent in older adults.	Respondents in the lowest two expenditure quintiles had the highest proportions of vision loss, hearing loss and DSL, and unmet needs for glasses and hearing aids whereas respondents in the highest two expenditure quintiles had the highest proportion of wearing glasses either regularly or from time to time.	Respondents with primary or under schooling had the highest proportion of vision loss, hearing loss, and dual sensory loss as well as the highest proportion of unmet needs for glasses and hearing aid/s; respondents with college or above degrees had the highest proportion of wearing a hearing aid, wearing glasses regularly, and those with secondary schooling had the highest proportion of wearing glasses from time to time.	NA	NA	NA	Respondents with a worse living standard than the average had the highest proportion of vision loss, hearing loss, DSL and unmet needs for glasses and hearing aids whilst respondents with a better living standard had the highest proportion of wearing glasses regularly and wearing hearing aids, and those with an average living standard had the highest proportion of wearing glasses from time to time;	it was evident that older people with poor/fair vision and/or hearing, or unmet needs for glasses or hearing aids had significantly lower participation in all of the social activities, compared to those with excellent, very good or good vision or hearing.	NA	NA
2574	Pineal	Dual Sensory Loss, Mental Health, and Wellbeing of Older Adults Living in China	Heine et al., 2019	China	Older adults residing in China, aged 60 years and over, experiencing DSL.	Data were collected from the China Health and Retirement Longitudinal Study Wave 2, 2013, using self-reported measures of vision and hearing loss, depression, and general wellbeing.	8268 participants with 4729 DSI.	60 years and older.	Age-related deafblindness	Dual Sensory Loss (DSL) is defined as "the acquired loss, in various degrees of severity, of both vision and hearing acuity, associated with aging and prevalent in older adults."	NA	that those with a lower attainment in education (with primary school or under, or second schooling) reported a significantly higher prevalence rate of DSL (59.1 and 55.2% respectively), whilst those with the highest level of education (college and above) reported the lowest prevalence of DSL	NA	NA	NA	Those respondents living rurally reported a significantly higher prevalence of DSL (61.0%) compared to respondents living in an urban environment (52.1%). The prevalence of DSL was 47.2%for older people with a relatively better living standard, and increased to a significantly higher rate (59.8%) for those with relatively worse living standard.	NA	NA	NA
119	Pineal	Dynamic associations between vision and hearing impairment and depressive symptoms among older Chinese adults	He et al., 2024	China	Older Chinese adults participating in the China Health and Retirement Longitudinal Study (CHARLS), which includes a broad age range of adults, particularly focusing on those aged 45 and older.	The study employed data from the CHARLS, which involved self-reported vision and hearing impairments and depressive symptoms assessed via the Center for Epidemiologic Studies Depression Scale (CESD-10).	8519 participants. Number with DSI not specified.	Aged 45 years and older.	Age-related deafblindness	Not specified	NA	NA	NA	NA	NA		NA	NA	NA
80	Pineal	Visual Impairment, Hearing Loss and Cognitive Function in an Older Population: Longitudinal Findings from the Blue Mountains Eye Study	Hong et al., 2016	Australia	Older adults residing in the Blue Mountains area, west of Sydney, Australia, aged 49 years and older.	The study utilized the Mini-Mental State Examination (MMSE) specifically adapted to exclude visually dependent tasks, along with assessments of visual acuity and hearing via audiometry across three follow-up visits spanning 15 years.	3654 participants at baseline with 2334 re-examined at the 5-year follow-up, with 93 having DSI, and decreasing numbers in subsequent follow-ups.	Aged 49 years and older.	Age-related deafblindness	Dual Sensory Impairment (DSI) was defined as the co-presence of visual impairment (best-corrected visual acuity <6/12) and hearing loss (pure-tone average >40 dB) detected at baseline.	NA	NA	NA	NA	NA		NA	NA	NA
3192	Pineal	Relational strain in close social relations among older adults with dual sensory loss	Hovaldt et al., 2019	Denmark	Older adults aged 50 years and older with acquired dual sensory loss (DSL), focusing on relational strains in close social relations.	Data were collected using a national cross-sectional questionnaire survey and an administrative database. The study also utilized multivariate logistic regression for analysis.	302 participants with 27 DSI.	Aged 50 years and older.	Age-related deafblindness	Dual sensory loss (DSL) was defined as a condition where both hearing and vision are concurrently impaired to a degree affecting access to information, communication, and mobility.	NA	NA	NA	NA	NA	NA	No associations between severity of dual sensory loss and excessive demands from or worries for children, other relatives, or personal support workers were found. Participants with total blindness and profound deafness had significantly higher odds for experiencing conflicts with children (odds ratio [OR] = 3.09, 95% confidence interval [CI] = [1.02, 9.38]) and the personal support worker (OR = 4.18, 95% CI = [1.23, 4.28]) compared to participants with residuals of both senses.	NA	NA
520	Pineal	A cross-sectional study of prevalence and correlates of self-harm and suicidal ideation in older adults with dual sensory loss	Hovaldt et al., 2022	Denmark	Adults aged 50 years or older with dual sensory loss (DSL), recruited from the national provider of services for people with DSL in Denmark.	Participants completed a national cross-sectional questionnaire survey that included questions related to thoughts of self-harm and suicide as well as physical, mental, and social wellbeing.	290 participants with DSL. (All participants have DSL).	Aged 50 years and older.	Age-related deafblindness	DSL is characterized by a significant reduction in both vision and hearing abilities, impacting daily functioning and access to information.	NA	NA	NA	NA	NA	NA	Not being involved in social participation was also associated with thoughts of SH or suicide (OR ¼ 2.30, 95% CI [1.16, 4.55]) compared to being involved in social participation.	NA	NA
376	Pineal	Association of Visual, Hearing, and Dual Sensory Impairment With Incident Dementia	Hu et al., 2022	United Kingdom	Community-dwelling individuals aged 40-69 years, specifically focusing on those with visual impairment (VI), hearing impairment (HI), and dual sensory impairment (DSI).	Data were collected from the UK Biobank study, utilizing visual acuity measurements, speech-reception-threshold information, and incident dementia identified through linked data to primary care, hospital admission records, and death registries.	113,511 participants with 730 DSI.	Aged 40-69 years at recruitment.	Age-related deafblindness	DSI was defined as having both visual impairment (VI worse than 0.3 LogMAR units) and hearing impairment (SRT of -5.5 dB or over).	NA	NA	NA	NA	NA	NA	NA	NA	NA
906	Pineal	Dual sensory impairment in older adults and risk of dementia from the GEM Study	Hwang et al., 2020	USA	Older adults aged 75 years and older, from the Ginkgo Evaluation of Memory Study, focusing on those with dual sensory impairment (DSI).	Data were collected through the Ginkgo Evaluation of Memory Study, which included self-reported measures of hearing and vision impairments and standardized criteria for dementia ascertainment.	2051 participants at baseline, 104 with DSI.	Aged 75 years and older.	Age-related deafblindness	DSI was defined based on self-reported impairments in hearing and vision.	NA	NA	NA	NA	NA	NA	NA	NA	NA

457	Pineal	Longitudinal Changes in Hearing and Visual Impairments and Risk of Dementia in Older Adults in the United States	Hwang et al., 2022	USA	Older adults aged 65 years and older participating in the Cardiovascular Health Study.	Data collection included longitudinal self-reported hearing and vision impairments, along with comprehensive dementia assessments using standardized criteria set by a multidisciplinary committee.	2927 participants with 120 DSI.	Aged 65 years and older.	Age-related deafblindness	Dual sensory impairment (DSI) was defined as having both hearing and vision impairments, which were self-reported.	NA	NA	NA	NA	NA	NA	NA	NA	NA
950	Pineal	Third-Party Impact of Dual Sensory Loss on Neuropsychiatric Symptom-Related Distress among Friends and Family	Kiely et al., 2020	Australia	Older adults aged 72 to 79 years, experiencing neuropsychiatric symptoms, and their close relations who were informants (spouse, child, friend, or other relatives).	Data was collected using the Personality and Total Health through Life (PATH) study. Informant distress was measured using the distress subscale of the Neuropsychiatric Inventory (NPI), and sensory functioning was assessed by visual acuity and self-reported hearing difficulties.	537 participant-informant dyads. DSI not specified.	Aged 72 to 79 years.	Age-related deafblindness	Not specified	NA	NA	NA	NA	NA	NA	NA	NA	NA
522	Pineal	Longitudinal Associations of Self-Reported Visual, Hearing, and Dual Sensory Difficulties With Symptoms of Depression Among Older Adults in the United States	Killeen et al., 2022	USA	Older adults aged 65 and older, participants of the National Health and Aging Trends Study (NHATS), assessing the impact of self-reported visual, hearing, and dual sensory difficulties on depressive symptoms.	The study utilized longitudinal data from the NHATS, involving self-reported assessments of visual and hearing difficulties and their association with depressive symptoms measured using the two-item Patient Health Questionnaire (PHQ-2).	7,593 participants 331 with DSI.	Aged 65 years and older.	Age-related deafblindness	Dual sensory difficulty is defined as a self-reported decrease in both hearing and visual functioning.	NA	NA	NA	NA	NA	NA	NA	NA	NA
10	Pineal	Impact of Concurrent Visual and Hearing Impairment on Incident Alzheimer's Disease: The LIFE Study	Sung-a Kim et al., 2024	Japan	Residents of five municipalities in Japan, participating in the Longevity Improvement & Fair Evidence (LIFE) Study, who had newly applied for long-term care needs certification between 2017 and 2022 and had no cognitive impairment upon application or AD diagnosis within the preceding six months.	Data collection involved the Basic Resident Register, long-term care needs certification questionnaire data, long-term care claims data, and medical care claims data.	14,186 participants 276 with DSI.	Adults aged ≥65 years and adults aged 40–64 years with specific diseases.	Age-related deafblindness	DSI is defined as the concurrent presence of visual impairment (VI) and hearing impairment (HI), with each being clinically diagnosed.	NA	NA	NA	NA	NA	NA	NA	NA	NA
293	Pineal	Association of Dual Sensory Impairment with Declining Physical Function in Community-Dwelling Older Adults	Kong et al., 2023	South Korea	Community-dwelling older adults aged 70–84 years, assessed for visual and hearing impairments.	The study utilized pure tone audiometry and visual acuity testing for sensory impairment assessment, and physical function was evaluated using handgrip strength tests and the Short Physical Performance Battery (SPPB).	2780 participants were included in the baseline cross-sectional analysis, and 2346 participated in the longitudinal follow-up. 187 with DSI.	Aged 70–84 years.	Age-related deafblindness	Not specified	NA	NA	NA	NA	NA	NA	NA	NA	NA
2118	Pineal	Self-reported dual sensory impairment and related factors: a European population-based cross-sectional survey	Leveziel et al., 2024	EUROPE: European Union (28 member states a	Individuals aged 15 years or more across 29 European countries, specifically focusing on adults aged 50 years or older for detailed analysis.	Data were collected using the European Health Interview Survey 2 (EHIS), employing standardized questionnaires covering demographic, socioeconomic, health determinants, and healthcare access.	296,677 individuals with 153,866 DSI.	Adults aged 50 years and older were specifically analyzed.	Age-related deafblindness	DSI was defined based on self-reported vision and hearing impairments, without using clinical measures to define sensory status.	Multivariable analyses showed that social isolation and poor self-rated health status were associated with DSI with ORs of 2.01 (1.77–2.29) and 2.33 (2.15–2.52), while higher income was associated with lower risk of DSI (OR of 0.83 (0.78–0.89). Considering country-level socioeconomic factors, Human Development Index explained almost 38% of the variance of age-adjusted prevalence of DSI.	NA	NA	NA	NA	NA	Multivariable analyses showed that social isolation and poor self-rated health status were associated with DSI with ORs of 2.01 (1.77–2.29) and 2.33 (2.15–2.52), while higher income was associated with lower risk of DSI (OR of 0.83 (0.78–0.89). Considering country-level socioeconomic factors, Human Development Index explained almost 38% of the variance of age-adjusted prevalence of DSI.	NA	NA
514	Aswen	Bidirectional associations	Liu et al., 2022	China	participants aged 45 years and older	A longitudinal study was conducted among 13,690 participants aged 45 years and older over four years. VL, HL, and DSL were identified through self-reporting, and depressive symptoms were assessed using a 10-item Center for Epidemiologic Studies Depression Scale. Multivariable Cox proportional hazards regression models were constructed to estimate the bidirectional associations of VL, HL, and DSL with depressive symptoms.	13690 participants, 1376 with DSI	45+	Not specified	sensory loss was identified through self-reports by the individual. Sensory loss	NA	NA	NA	NA	NA	NA	NA	NA	NA
365	Aswen	Sensory impairment and co	Liu et al., 2023	China	10,895 older adults aged 65 and above	Based on the 2018 Chinese Longitudinal Healthy Longevity Survey, a total of 10,895 older adults aged 65 and above were selected as samples for research. Anxiety, depressive symptoms and Cognitive function were evaluated by the Generalized Anxiety Disorder, the Center for Epidemiologic Studies Depression (CES D10) and the Chinese version of modified Mini Mental State Examination scales, respectively. Sensory impairment was assessed from self reported vision and hearing functions. Multiple linear regression and SPSS Macro PROCESS were used for statistical analysis.	10895 participants, 1003 with DSI	Age 65+; mean age 79.84 ± 9.909	Not specified	Hearing impairment referred to complete or partial loss of the ability to hear. In the process of questionnaire survey, interviewers evaluated the hearing function by assessing whether the respondents can clearly hear the interviewed content. Four options are available: (1) Yes, without hearing aid; (2) Yes, but needs hearing aid; (3) Partly, despite hearing aid; (4) No. Respondents were assessed as having HI if they could not hear clearly what the interviewers said or used a hearing aid to hear all or part of it (i.e., the option 2–4). To assess VI, the respondents were asked whether they, after taking off correction (e.g., glasses), could see a break in the circle on the cardboard sheet when lit by a flashlight and distinguish where the break is located. The circle was positioned in 1 m away from all respondents. There were four options: (1) can see and distinguish; (2) can see only; (3) cannot see; (4) blind. The respondents were identified as having VI if they were unable to distinguish the direction of the break, could not see the circle or were blind. Respondents were regarded as DSI if they had both vision and HI.	NA	NA	NA	NA	NA	NA	NA	NA	NA
182	Aswen	Association of changes in s	Li et al., 2024	China	10,050 aged >45 with visual impairment and hear	The 10-item Center for Epidemiologic Studies Depression Scale (CES-D10) was used to assess depressive symptoms. Total scores and clinically significant depressive symptoms (CES-D10 scores ≥ 10) were used as outcomes and analyzed using generalized estimating equations with identity link and logit link functions.	10050 with vision and hearing impairment witho	people aged ≥ 45 years; 58.9 years (	Not specified	Vision impairment (VI) and hearing impairment (HI). We used two questions, one on near vision and one on distance vision, to fully assess the vision status of the participants. We asked the respondents how comfortable they felt while recognizing their friends across the street and while reading newspapers and other commonly printed materials when wearing glasses or corrective lenses if required. To determine HI, we asked the participants to report their hearing status (when wearing hearing aids if required). All three questions were scored on a 5-point scale, with 1 = excellent and 5 = poor. We defined participants as having "good" hearing or vision status if they answered "excellent," "very good," or "good" to one question on hearing or both questions on vision. We defined participants as having "poor" hearing or vision status if they answered "fair" or "poor" to the hearing question or one of the vision questions. This self-reported assessment and classification method has also been used in previous studies (Chou & Chi, 2004; Han et al., 2019).	NA	NA	NA	NA	NA	NA	NA	NA	NA
915	Aswen	Prevalence, diagnoses and	Lundin et al., 2020	Sweden	Persons aged >65	cross-sectional medical chart review	101 with DSL												

420	Aswen	Experiences of rehabilitatic	Lundin et al., 2022	Sweden	older adults aged >65 with HL and VI (DSL)	qualitative explorative design - study of lived experiences through semi-structured interviews	20 older adults >65 with DSL	aged over >65; mean age NR	not specified	Participants fulfilled the objective standardized measurements for both vision	NA	NA	NA	NA	NA	NA	NA	NA	The participants provided rich experiences about how they were maintaining and regaining functions through their rehabilitation services. The participants described that these interventions were significant for them to continue with their life-roles and activities in daily life. The participants also expressed the importance of meeting others in a similar situation and developing strategies to master their situation. Further, the participants expressed that the accessibility of rehabilitation services and professional encounters and attitudes impacted their experiences and the outcome of the rehabilitation. This notion was discussed in terms of factors that facilitated or hindered the participants. Despite the DSL, the participants' focus was mostly on
1389	Aswen	Gender-Specific Associatic	Lyu 2018	South Korea	nationally representative population of age >65	The measure of cognitive impairment was derived from the Korean version of the Mini-Mental State Examination (K-MMSE) that had a maximum score of 30 points (Cronbach's alpha: 0.880).	3831 (2167 female (134 with DSI); 1664 male (!	age 65+; mean female age 73.9 (SD	Not specified	Both self-reported status of eyesight and hearing were assessed with a five-p	NA	NA	NA	NA	NA	NA	NA	NA	NA
686	Aswen	Depression, combined visu	Marmamula et al., 2021	India	participants aged 60+ in residential care	Questionnaire administered through interview; Hindi Mini Mental State Examination questionnaire, Patient Heatlh Questionnaire (PHQ-9), Hearing Handicap Inventoty for the Elderly Screening (HHIE)	50 with DSI	age over 60; mean age 74.2 (SD 8.2)	Not specified	Visual impairment (VI) was defned as presenting visual acuity worse than 6/18 in the better eye.Hearing impairment (HI) was defned as HHIE Score greater than ten as recommended by previous authors. Dual Sensory Impairment (DSI) was considered to be present if a participant had both VI and HI.	NA	NA	NA	NA	NA	NA	NA	NA	NA
1001	Aswen	Impact of sensory impairm	Maruta et al., 2020	Japan	older adults without dementia	Retrospective review of long-term care insurance certification data	2190 older adults; 295 with DSI	78.9 (SD 6.1)	Not specified	VI and HI were determined based on a previous study,17	NA	NA	NA	NA	NA	NA	NA	NA	NA
1345	Aswen	Associations between sensory loss and social networks, participation, support, and loneliness	Mick et al., 2018	Canada	participants in the Canadian Longitudinal Study o	Cross-sectional population-based study.	21241 participants	age 45-89;	Not specified	Self-reported hearing was determined using the following multiple-choice item: "Is your hearing, using a hearing aid if you use one ..." Participants were classified as having hearing loss if they responded "fair" or "poor, nonexistent, or deaf" (vs "good," "very good," or "excellent"). Self-reported vision was determined using the following multiple-choice item: "Is your eyesight, using glasses or corrective lenses if you use them ..." Participants were classified as having vision loss if they responded "fair" or "poor, nonexistent, or blind" (vs "good," "very good," or "excellent"). Individuals were considered to have dual sensory loss if they had both hearing loss and vision loss defined according to these criteria	NA	NA	NA	NA	NA	NA	Social network diversity: Hearing loss was not associated with social network diversity. Vision loss was independently associated with lower social network diversity among men but not women (P = .022). Dual sensory loss was significantly associated with reduced social network diversity among 65- to 85-year-olds but not 45- to 64-year-olds (P=.037). Social participation: Vision loss, but not hearing loss, was independently associated with reduced social participation (Table 4). Dual sensory loss was also associated with low social participation, but only in the 65- to 85-year-old age group (P=.032). Availability of social support: Hearing, vision, and dual sensory loss were each independently associated with lower overall availability of social support and with nearly all domains of social support. Loneliness: Hearing, vision, and dual sensory loss were each independently associated with	NA	NA
1830	Aswen	Vision and hearing impairm	Mitoku et al., 2016	Japan	Subjects of the study were community-dwelling e	population-based cohort study used administrative health care data with a mean follow-up period of 4.7 years	1754 participants	mean age for women 82.42 (6.95); f	Not specified	Vision at baseline was determined according to five levels: "normal sight," "able to see a visual acuity chart at a distance of 1 m," "able to see a visual acuity chart in front," "very little sight," and "indeterminable due to communication difficulty." For analysis, "able to see a visual acuity chart at a distance of 1 m" and "able to see a visual acuity chart in front" were merged to form one value—"able to see an object near the front of the eyes." Hearing at baseline was determined according to five levels: "normal hearing," "barely hear normal conversation," "barely hear loud conversation," "barely hear," and "indeterminable due to communication difficulty." For analysis, "barely hear normal conversation" and "barely hear loud conversation" were merged to form one value—"able to hear loud conversation."	NA	NA	NA	NA	NA	NA	NA	NA	NA
1114	Aswen	Trajectories of Limitations	Mueller-Schotte et al., 2019	Netherlands	Participants were eligible if they were 60 years of age or older and had complete baseline data on all variables relevant to this research question (no sensory loss, hearing loss, vision loss, dual sensory loss)	longitudinal population-based study; Data of the Dutch "The Older Persons and Informal Caregivers Survey Minimum Dataset" (TOPICS-MDS), a dynamic public access database of 42 research studies, were used	all participants (9319); dual sensory loss (1693	age over 60; mean age 78.58 (SD 6.(	Not specified	Self-reported HL and VL were assessed by asking the participant "Place a tick	NA	NA	NA	NA	NA	NA	The age effect on difficulties preparing a meal was highest for DSL (OR = 1.66 [95% CI = 1.56; 1.77]) and lowest for VL (OR = 1.38 [95% CI = 1.38; 1.46]). Point estimates for HL and NSL lay between DSL and VL.	NA	NA
1639	Aswen	Problems identified by dua	Roets-Merken et al., 2017	Netherlands	Dual sensory impaired older adults in the intervention group (n = 47, age range 82–98) were invited by a familiar nurse to identify the problems they wanted to address	A qualitative study was conducted in parallel with a cluster randomized controlled trial. Data were taken from the semi-structured intervention diaries in which nurses noted the older adults' verbal responses during a five-month intervention period in 17 long-term care homes across the Netherlands	47 participants with dual sensory imapirments	age 55+; mean age 90.8 (SD 4.4)	Not specified	(1) aged 55 or over, (2) a hearing impairment of PTA >40 dB (best ear) and a visual impairment with a best-corrected visual acuity of <0.3 diopter or with a visual field of <30°, measured using the criterion standards for hearing and visual impairment and (3) written informed consent.	NA	NA	NA	NA	NA	NA	NA	NA	The theme participation reflects three c
908	Aswen	Dual Sensory Impairment a	Shakarchi et al., 2020	USA	This study included participants from the Health a	Cross-sectional analysis of the Health and Retirement Study 2006 and 2008 surveys, a US population-based survey that included noninstitutionalized adults 51 years and older. Analyses were weighted to account for complex sample design and differential nonresponse. Data were analyzed between October 2019 and November 2019.	NSI (neither sensory impairment) 8636; VI alon	aged 51+; 70.6 (11.8)	Not specified	Sensory impairment was assessed using patient-reported functional measures. Visual ability was assessed on a single item: "Is your eyesight excellent, very good, good, fair, or poor using glasses or corrective lens as usual?" Hearing ability was similarly assessed on a single item: "Is your hearing excellent, very good, good, fair, or poor?" Participants who indicated using a hearing aid during the interview or any prior interview were asked to rate their hearing "using a hearing aid as usual." Impairment was defined as reporting fair or poor sensory ability on the respective question(s). Dual sensory impairment was defined as having concurrent VI and HI.							participants with VI alone (β = 0.07; 95% CI, 0.02-0.13) and HI alone (β = 0.07; 95% CI, 0.02-0.11) had higher discrimination scores (meaning greater levels of discrimination) compared with the NSI group, **but this difference was greatest for those with DSI (β = 0.23; 95% CI, 0.16-0.29)**. The prevalence of frequent discrimination was higher by 38% (95% CI, 20%-58%) for VI alone, by 21% (95% CI, 3%-43%) for HI alone, and by 72% (95% CI, 47%-100%) for DSI when compared with NSI		

2114	Aswen	The association between a	Sheng et al., 2023	China	Chinese residents aged 45 years and above were recruited from the China Health and Retirement Longitudinal Study 2011–2018, using stratified random sampling. T	Logistic regression and restricted cubic splines models were used for this cross-sectional and longitudinal analysis	6812 overall participants; 3168 with DSI	60.81 ± 8.43	Not specified	Participants were asked to rate their vision (including far and close vision) and hearing status (including corrected hearing) on a scale from 1 (Excellent) to 5 (Poor). A rating of "Fair" or "Poor" for vision or hearing was classified as "SI" (Zhang et al., 2022). Single sensory impairment (SSI) was divided into SVI and SHI, which were mutually exclusive. Simultaneous occurrence of visual impairment and hearing impairment was considered as DSI. The progression of SI status was assessed from the 2011 baseline survey (the starting point) to the wave four survey in 2018 (the endpoint). To ensure an adequate sample size in every progression group, we employed the broader category of SSI rather than distinguishing between SVI and SHI. Progression groups were defined as follows: 1) maintained no SI; 2) no SI to SSI; 3) no SI to DSI; 4) maintained SSI; 5) SSI to DSI; 6) maintained DSI.	More than half of the ACE types were associated with increased odds of DSI, except for emotional abuse, household substance abuse or incarceration, neglect, and adverse outside-family environment.	Having ≥4 adverse childhood events (ACEs) was positively associated with higher odds for DSI (OR = 1.57, 95 % CI = 1.20–2.06) than the odds for no SI. Comparing to having no ACEs, having at least four ACEs was positively associated with higher odds of progression of SI from SSI to DSI (OR = 1.71, 95 % CI = 1.03–2.84) and maintained DSI (OR = 2.23, 95 % CI = 1.37–3.65) compared to maintained no SI							
1572	Aswen	Depressive and anxiety syr	Simning et al., 2017	USA	US Medicare beneficiaries with auditory and vision impairment	The NHATS in-person interview occurs annually and is administered in English and Spanish; large print cards are used to help older adults answer the interview questions ("National Health & Aging Trends Study," 2017). Among these 7,507 participants, 547 proxy interviewees were questioned when the sampled NHATS older adults could not respond in person to the interview questions (e.g., due to a physical or cognitive impairment)	7,507 older adults from the National Health and Aging Trends Study, a nationally representative sample of United States Medicare beneficiaries; 125 with DSI	Age 60+; mean age not reported	Not specified	Auditory and vision impairment were determined by self-report, and depressive and anxiety symptoms were evaluated by the PHQ-2 and GAD-2, respectively.	NA	NA	NA	NA	NA	NA	NA	NA	NA
364	Aswen	Self-Reported Dual Sensor	Smith et al., 2022	USA	The NHIS is an ongoing household health survey sampling 35,000 households each year. By design, the survey is representative of the community-dwelling population living in the United States.	Cross-sectional analysis of 9,899 community-dwelling respondents aged 60+ years without dementia or depression in the 2019 National Health Interview Survey. Participants self-reported difficulty remembering or concentrating, seeing even when wearing corrective lenses, and hearing even when using a hearing aid. We defined SCCs and sensory impairment for each mode as reporting at least some difficulty. We categorized sensory impairment into no sensory impairment, vision impairment only, hearing impairment only, and DSI. We then estimated weighted prevalence ratios (PRs) of SCCs by impairment category.	overall 9899; 594 with DSI	age over 60; mean age 73 (8)	Not specified	We ascertained self-reported hearing impairment via the survey question, "Do you have difficulty hearing [even when using your hearing aid]?" and self-reported vision impairment via the question, "Do you have difficulty seeing [even when wearing glasses or contact lenses]?" We dichotomized self-reported hearing into hearing impairment (response indicating "Some difficulty," "A lot of difficulty," or "Cannot do at all") and no hearing impairment (i.e., response indicating "No difficulty"). We further dichotomized self-reported vision into visual impairment (i.e., response indicating "Some difficulty," "A lot of difficulty," or "Cannot do at all") and no visual impairment (i.e., response indicating "No difficulty"). We classified participants who reported both hearing and visual impairments as having DSI. We then categorized participants into mutually exclusive groups of no sensory impairment, hearing impairment only, vision impairment only, and DSI.	NA	NA	NA	NA	NA	NA	NA	NA	NA
441	Aswen	Association of Dual Sensory Impairment with Cognitive Decline in Older Adults	Tomida et al., 2022	Japan	Participants were recruited from a sub-cohort of t	The participants were recruited from a sub-cohort of the National Center for Geriatric Gerontology-Study on Geriatric Syndromes (NCGG-SGS) conducted by the National Center for Geriatrics and Gerontology. Logistic regression analysis was used to evaluate the relationship between the presence of HI and VI and the severity of MCI, and functional decline in each cognitive domain	We included 4,471 community-dwelling older a	age: 75.9 ± 4.3 years	Not specified	The HI and VI were identified using a self-report questionnaire. Cognitive and other parameters were also assessed by trained staff	NA	NA	NA	NA	NA	NA	NA	NA	NA
639	Abi	Association of dual sensory impairment with changes in life space: A longitudinal study with two year follow-up	Tomida et al., 2022	Japan	adults aged ≥70 years as of January 1, 2013, residing in Midori-ward, Nagoya, Aichi, and did not require long-term care needs or support at the start of the study	Hearing Handicap Inventory for the Elderly and Adults-Screening (HHIE-S) Version<br>Visual Function index 14 (VF-14)<br>Active Mobility Index<br>questionnaire that comprised 16 questions regarding instrumental ADL (IADL), cognitive activity, social activity, and productive activity<br>Geriatric Depression Scale (GDS)	4214 participants; with 476 participants with DBI	aged over 70 years	dual sensory impairment	defined as concurrent VI and HI	NA	NA	NA	NA	NA	NA	In a comparison of responses to questions on 16 ADLs grouped into four categories: IADL, cognitive activities, social activities, and productive activities, SSI showed significantly lower performance on IADL, driving a car, and using a map to go to unfamiliar places; cognitive activities: culture lessons and using per- sonal computers; social activities: giving someone helping hand; and productive activities; helping your family and friends (P < 0.01). In the DSI, significant differences were found in all items except for the pres- ence of work in productive activities (P < 0.01).	NA	NA
180	Abi	Longitudinal Analysis of Social Isolation and Cognitive Functioning among Hispanic Older Adults with Sensory Impairments	Tanner et al., 2023	USA	557 older Hispanic adults that participated in the National Health and Aging Trends Study (NHATS), a nationally representative study of Medicare beneficiaries in the United States aged 65 and older	Cognitive measures: cognitive measures collected in NHATS corresponding to orientation, execu- tive function, and learning/memory<br>self-reported vision impairment (VI), hearing impairment (HI), and dual sensory impairment (DSI) using 3 variables<br>Social isolation scale	557 participants	aged 65 and older	dual sensory impairment	Dual sensory impairment (DSI) consists of concurrent vision and hearing loss	NA	NA	NA	NA	NA	NA	Contrary to our expectations, neither VI nor HI were significantly associated with so-<br>cial isolation concurrently or across time. Unlike VI and HI alone, DSI was associated<br>with higher social isolation one year later in both Model 1<br><br>DSI was negatively associated with orientation and learning/memory.	NA	NA
540	Abi	Dual Sensory Impairment as a Predictor of Loneliness and Isolation in Older Adults: National Cohort Study	Wang et al., 2022	China	Older adults who participated in the China Health and Retirement Longitudinal Study (CHARLS)	data used in this study were from the 2011, 2013, 2015, and 2018 waves of the China Health and Retirement Longitudinal Study (CHARLS).	3069 older adults; 1685 reported DSI	aged 60 and older	dual sensory impairment	the co-occurrence of VI and HI	NA	NA	NA	NA	NA	Participants in rural areas were more likely to report DSI (1442/2539, 56.79% vs 243/530, 45.85%; P<.001) and loneliness (847/2539, 33.36% vs 116/530, 21.89%; P<.001) than those in urban areas.	As shown in Figure 1, among older adults in urban areas, participants with VI only (OR 2.44, 95% CI 1.57-3.80; P<.001), HI only (OR 2.47, 95% CI 1.41-4.32; P=.002), and DSI (OR 1.88, 95% CI 1.24-2.85; P=.003) were more likely to experience loneliness compared with participants without SIs. mong older adults in rural areas, participants with DSI were more likely to experience loneliness compared with those without SIs	NA	NA
1076	Abi	The Association between Dual Sensory Impairment and Hospital Admission in California Medicare Beneficiaries	Wong et al., 2022	USA	resi- dence in California during 2015, age 65 years or older, enrolled in Medicare part A and part B, and had at least one part B claim in 2015.	trospective cross-sectional study was conducted using administrative data from all U.S. California Medicare beneficiaries in 2015. Standard Analytic Files of Medicare Inpatient Claim, Part B Carrier Claim, and Master Beneficiary Summary File (MBSF) data files from the calendar year of 2015 were obtained from the Centers for Medicare & Medicaid Services (CMS). Diagnosis codes based on the International Classification of Disease – 9th and 10th Edition (ICD-9 and ICD −10),21,22 and hospitalization status were pro- vided in Medicare Standard Analytic Files.	2,574,641 California Medicare beneficiaries 3,055 had DSI	65 and older	dual sensory impairment	NA	NA	NA	NA	NA	NA	NA	NA	NA	NA
782	Abi	The longitudinal effect of sensory loss on depression among Chinese older adults	Xie et al., 2021	China	6353 Chinese older adults aged 60 years and over	China Health and Retirement Longi- tudinal Study (CHARLS)			dual sensory loss	defined as a combined loss of both hearing and vision	NA	NA	NA	NA	NA	NA	NA	NA	NA
40	Abi	Sensory impairment and depressive symptoms among older adults before and during the COVID-19 pandemic	Xu et al., 2024	USA		Health and Retirement Study (HRS)	1300 with DSI	Aged 50 and above	dual sensory impairment	problems with both vision and hearing	NA	NA	NA	NA	NA	NA	DSI group was more likely to have any ADL limitations	NA	NA

1834	Abi	Dual Sensory Impairment and Cognitive Decline:The Results From the Shelter Study	Yamada et al., 2015	EUROPE: (Czech Republic, England, Finland, France, Germany, Israel, Italy, and the Netherlands)	nursing home residents from the Services and Health for Elderly in Long TERm Care study	interRAI LTCF	122 with DSI	Not reported but average is 88.7 at baseline	dual sensory impairment	concurrent vision and hearing impairment	NA	NA	NA	NA	NA	NA	The greatest cognitive decline was seen in residents with DSI and social disengagement: 1.87 (95% CI = 1.24:2.51) point decrease in cognitive function over 1 year. mong socially disengaged residents, the cognitive decline that residents with DSI experienced was significantly greater than that of residents with no impairment [differences between the groups was 1.07 (95% CI = 0.40:1.75)] and marginally greater than that of residents with single impairment [0.68 (95% CI= −0.06:1.42)], while it was not among socially engaged residents. Absence of social engagement was associated with a greater cognitive regardless of the status of sensory impairment, with the largest difference being observed in residents with DSI.	NA	NA
1832	Abi	Joint Associations of Dual Sensory Impairment and No-Activity Involvement With 1-Year Mortality in Nursing Homes: Results From the SHELTER Study	Yamada et al., 2015	EUROPE: Czech Republic, England, Finland, France, Germany, Israel, Italy, and the Netherlands	nursing home residents from the Services and Health for Elderly in Long TERm Care study	interRAI LTCF	122 with DSI	Not reported but average is 88.7 at baseline	dual sensory impairment	concurrent vision and hearing impairment	NA	NA	NA	NA	NA	NA	DSI residents without involve- ment in activities had a higher risk of mortality (HR = 1.51, 95% CI = 1.09–2.10), equivalent to an additional 137 (95% CI = 10 –264) deaths per 1,000 person-years.	NA	NA
542	Abi	he Longitudinal Association of Late-Life Visual and Hearing Difficulty and Cognitive Function: The Role of Social Isolation	Yorgason et al., 2022	United States	National Health and Aging Trends Study (NHATS) is a nationally representative panel study of Medicare benefi- ciaries' age ≥ 65 years	National Health and Aging Trends Study	6338 TOTAL; 140 DSI	65 and older	dual sensory impairment/dual sensory difficulty	Not specified	NA	NA	NA	NA	NA	NA	Baseline DSD was associated with all three concurrent cognitive measures through social isolation (learning/memory: 11% of total ef- fect, p < .01; orientation: 6% of total effect, p < .01; executive function: 6% of total effect, p < .01) and was significantly associated longitudinally with learning/memory (10% of total effect, p < .05) 1 year later through Round 6 social isolation.	NA	NA
2169	Abi	Association of Sensory Impairment With Institutional Care Willingness Among Older Adults in Urban and Rural China: An Observational Study	Zhang et al., 2023	China	participants of sixth National Health Service Survey of Shandong Province, China, in 2018.	ational Health Service Survey (NHSS), a cross-sectional large-scale nationally represen- tative sampling survey launched by the National Health Commission of China every 5 years since 1993	8 583 individual	aged ≥60 years	dual sensory impairment	Not specified	NA	Compared to other groups, older adults with DSI were more likely to be female (urban areas: 56.5%, p < .001; rural areas: 56.4%, p < .001), older (urban areas: 72.9 ± 8.4, p < .001; rural areas: 71.2 ± 7.4, p < .001), illiterate (urban areas: 38.4%, p < .001; rural areas: 47.9%, p < .001), single (urban areas: 26.0%, p < .001; rural areas: 23.9%, p < .001), and had a higher rate of unemploy- ment (urban areas: 57.5%, p < .001; rural areas: 64.2%, p < .001).	Compared to other groups, older adults with DSI were more likely to be female (urban areas: 56.5%, p < .001; rural areas: 56.4%, p < .001), older (urban areas: 72.9 ± 8.4, p < .001; rural areas: 71.2 ± 7.4, p < .001), illiterate (urban areas: 38.4%, p < .001; rural areas: 47.9%, p < .001), single (urban areas: 26.0%, p < .001; rural areas: 23.9%, p < .001), and had a higher rate of unemploy- ment (urban areas: 57.5%, p < .001; rural areas: 64.2%, p < .001).	NA	NA	Of the sample with DSI, 7.0% had the willingness of institutional care, and 6.7% had the willingness of institutional care of the sample with VI only. In rural areas, 9.9% older adults without SI, 8.0% older adults with HI only, 7.9% older adults with VI only, and 7.0% older adults with DSI preferred institutional care.	NA	NA	NA
392	Abi	Longitudinal relationship between sensory impairments and depressive symptoms in older adults: The mediating role of functional limitation	Zhang et al., 2021	China	Medicare benefi- ciaries' age ≥ 65 years	analysis of National Health and Aging Trends Study (NHATS) is a nationally representative panel stud	4130 older adults; 58.6%, had DSI	60 and older	dual sensory impairment	co occurrence of VI and HI.	NA	NA	NA	NA	NA	NA	NA	NA	NA
**TOTAL**	**69**										**6**	**6**	**3**	**0**	**1**	**5**	**21**	**0**	**9**

	Disability
	Social Inclusion and Non-Discriminatio
	Gender
	Access to Affordable Health
Total	Income and Social Protection
	Education
	Housing, Basic Amenities, and the Envi
	Unemployment and Job Security
	Race
	Food Insecurity
	Working Life Conditions
	Structural Conflict
	Indigenous Status

				KEY TAKEAWAYS FROM ARTICLE		
**DISABILITY**	**GENDER**	**RACE**	**INDIGENOUS STATUS**	**SIGNIFICANT FINDINGS**	**RECOMMENDATIONS OF ARTICLE**	**OTHER KEY POINTS**
Copy and paste the section of the results section of the article that examines individuals with disability. In all of the articles to be extracted, they should include individuals with DSI. However, this section is focused on other disabilities such as depression, dementia/cognitive decline, physical disabilities. If not discussed, write NA.	*Copy and paste the section of the results section of the article that examines gender impacts of deafblindness. For instance, the article can discuss about females with deafblindness facing greater issues in acessing social services than males with deafblindness or females without disability. If not discussed, write NA.*	*Copy and paste the section of the results section of the article that examines racial mpacts of deafblindness. For instance, the article can discuss about*	*Copy and paste the section of the results section of the article that examines Indigenous status in individuals with deafblindness. For instance, the article can discuss about Indigenous people with deafblindness experience. If not discussed, write NA.*	*This can be found in the discussion paragraph where the author talks about key findings from the article as it relates to DSI and SDH. Please keep this concise to 4-5 sentences. If there are lots of important findings, please include the page number. If you are unsure, please write "Unsure." If you have more to include, please refer to the page number.*	*This can typically be found after key points and often begins with the lines "It is recommended" or "It is suggested." Recommendations/ suggestions are typically made at the policy, program, and/or research level. Please keep this concise to 4-5 sentences. If there are lots of significant recommendations, please include the page number. If you are unsure, please write "Unsure."*	*This is a section for you, the reviewer, to explain anything that you believe is significant for the research team. If you have nothing to include, please write "NA." If you are unsure, please write "Unsure."*
NA	The only significant associations with sex were found for the Informal Support CAP, the Pain scale, and the item "length of time alone during the day", with females showing poorer results	NA	NA	In most of the areas assessed (cognition, communication, ADLs, IADLs, mental health, social relationships), the majority of participants in all sensory impairment groups showed high functioning and were not at risk for health problems in the immediate future, as measured with the interRAI CHA. Our findings highlight that despite having sensory losses, community-dwelling older adults who receive rehabilitation services are experiencing overall good health and a high level of independence.	Specific comparison group (HL or VL) influences if DSL shows poorer outcomes	Large takewaway: DSL not always assocaited with worse results compared to single impairment and depends more on task and comparsion group
NA	NA	NA	NA	Five factors were identified within this theme, which were perceived to influence the pharmaceutical care of older patients with sensory impairment: community pharmacy personnel's awareness of the sensory impairment; community pharmacy personnel's perception of older people with sensory impairment; relying on carer support; pharmacy layout and physical access and organisational factors	Participants in the study suggested generic and focussed solutions for improving pharmaceutical care that included raising awareness of the difficulties faced by patients with sensory impairment as a result of communications and environmental barriers and good examples of practice where the pharmaceutical care needs of this patient population had been met. Most interviewees discussed training needs in relation to providing appropriate pharmaceutical care for this patient population. A range of training needs was identified for pharmacists as well as other pharmacy personnel - counter assistants, technicians and drivers e and included: skills in identifying people with sensory impairment; enhanced communication techniques, for example, interpersonal skills on how to approach someone with a sensory impairment without patronising them; training in sign language; and use of sensory aids such as hearing loops. Some interviewees described specific approaches to help with providing pharmaceutical care for older people with sensory impairment, including: simplifying complex dosing schemes; counselling at home; notes on sensory needs in patient files; medicines administration records chart; consulting the patient about what could help them and providing hearing tests in the pharmacy. One participant also suggested having a referral system to specialist pharmacists trained to communicate with people with hearing or sight impairment.	
NA	NA	NA	NA	The key themes that emerged included barriers and facilitators in relation to ordering and accessing medicines and in terms of managing medicines appropriately. Non-identification of sensory impairment or non-disclosure as well as various communication and accessibility challenges were perceived as possible safety hazards.	(1) educating the patient about their drug regimen and their medical condition, (2) implementing compliance-improving strategies such as drug reminder charts and (3) rationalising and simplifying drug regimens in collaboration with the patient's general practitioner. Involving patients in service improvement and health professional education and training plays a key part in the redesign of healthcare delivery. Community pharmacy personnel need to provide information in suitable, alternative formats, minimise (where possible) and explain changes in medication appearance to service users, and perform regular medicine reviews using a patient-centred approach to provide safe and effective pharmaceutical care. Community pharmacy personnel identified a need for evidence-informed education and training in the provision of pharmaceutical care to older people with sensory impairment	
NA	NA	NA	NA	Our study results indicate that whilst some services are available in most care homes for ear and eye care across England, others are not as frequently implemented or that staff are unsure of whether such services are available or not. This highlights the underuse of the certain services and implies patchy implementation of the practices which could significantly benefit residents hearing and vision capabilities and improve mental and physical health.	Best practice for ear and eye care in care home residents can be improved, and this evidence identifies specifically which practices should be targeted, including screening tools, annual hearing tests conducted by external services and having access to various devices. In addition, staff knowledge shows room for improvement, with the majority of staff wanting more information on how to care for both hearing and vision in their residents. This study confirms the need for better support for care home staff so that they can provide optimum ear and eye care including services provided by others external to the home. It also identifies a need for further research to explore how quality services can be both provided and sustained over time. Due to the limited evidence, the implementation of mentioned practices should be evaluated by research on the effectiveness and benefit for residents with regard to hearing and vision. This could also focus on the improvement of mental and physical health and residents' quality of life.	
After covariate adjustment, DSI was associated with 2.25 times the risk of incident mobility difficulty (95% CI: 1.47, 3.43). VI was marginally associated with risk of incident mobility difficulty (HR=1.55, 95% CI: 0.96, 2.50), but HI was not significantly associated with risk of incident mobility difficulty (HR=1.26, 95% CI: 0.83, 1.91) (Table 2). The RERI was 0.43 (95% CI: −0.38, 1.24), and the SI was 1.53 (95% CI: 0.00, 3.06). The estimate when combining HI and VI was 1.96 (95% CI: 0.40, 3.51), suggesting that DSI did not contribute more than the sum of the individual sensory impairments. Adding hearing aid use to the model did not change inferences. We examined the associations of DSI with incident mobility difficulty after excluding prevalent dementia cases four years after study initiation, and our inferences remained the same. HI was not significantly related to incident mobility difficulty (HR=1.26, 95% CI: 0.81, 1.96). VI was marginally associated with increased risk of incident mobility difficulty (HR=1.51, 95% CI: 0.91, 2.51). DSI was associated with increased risk of incident mobility difficulty (HR=2.48, 95% CI: 1.58, 3.90). In [illegible]	). For both males and females, DSI, not HI and VI, was associated with increased risk of mobility difficulty (males: HR=1.77, 95% CI: 1.03, 3.04; females: HR=3.26, 95% CI: 1.64, 6.47). For males, DSI was associated with increased risk of incident ADL difficulty (HR=1.79, 95% CI: 1.06, 3.04), but HI and VI were not associated with incident ADL difficulty. For women, DSI (HR=3.08, 95% CI: 1.62, 5.86) was associated with increased risk of incident ADL difficulty	In whites, DSI was associated with increased risk of mobility difficulty (HR=1.84, 95% CI: 1.07, 3.16). In Blacks, both VI (HR=2.30, 95% CI: 1.14, 4.67) and DSI (HR=3.38, 95% CI: 1.70, 6.70) were associated with increased risk of mobility difficulty. Supplementary Table 1 shows that the DSI increases risk of incident mobility difficulty (RR=1.94, 95% CI: 1.30, 2.90), but not in excess risk (RD=0.016, 95% CI:−0.020, 0.052), after covariate adjustment. In whites, DSI was associated with increased risk of incident ADL difficulty (HR=2.02, 95% CI: 1.19, 3.43), while in Blacks, DSI (HR=3.00, 95% CI: 1.57, 5.73) was associated with increased risk of ADL difficulty. Supplementary Table 1 shows that DSI increases risk of incident mobility difficulty (RR=2.00, 95% CI: 1.36, 2.95), but not in excess risk (RD=0.004, 95% CI: −0.032, 0.039), after covariate adjustment.		DSI is associated with increased risk of incident mobility and ADL difficulty. Rehabilitation and adaptive environmental changes for individuals living with DSI may be important to maximize mobility and daily function.	Given the high prevalence of DSI among older adults, few people who need them adopt hearing aids, due to costs and access to care, but this could be changing based on the 2017 OTC Hearing Aid Act to make hearing devices more readily available for public use. Besides hearing aids, cataract surgery may reduce falls42 and lower dementia incidence since VI is an important risk factor of fall and injury. Each sensory impairment should be considered in the risk of incident mobility and ADL difficulty. Furthermore, audition and low vision rehabilitation, hearing aid use, and environmental modifications at home and in society can enhance mobility as people age with sensory impairments. Rehabilitation can introduce compensatory strategies, modifications in environment, and use of assistive tools if needed. These can promote better self-esteemed, self-confidence, postpone cognitive decline, leading to independent living for longer periods of time44, 45 and better quality of life46. Additionally, previous studies have shown the link between mobility and ADL difficulty increasing risk of mortality among older adults47–49, so modifying the onset of DSI may delay mobility and ADL difficulty, which in turn, could decrease the risk of mortality	

NA	NA	NA	NA	DSI was associated with increased risk of being chronically depressed (Risk Ratio, RR = 1.99, 95 % Confidence Interval, CI: 1.25, 3.17), not mild/moderate increasingly depressed (RR = 1.25, 95 % CI: 0.91, 1.71). DSI had increased risk of being mild/moderate increasingly anxious (RR = 1.60, 95 % CI: 1.16, 2.19) and chronically anxious (RR = 1.86, 95 % CI: 1.05, 3.27) groups, as compared to no impairments. Hearing impairment was associated with being mild/moderate increasingly anxious (RR = 1.34, 95 % CI: 1.01, 1.79). No other associations were found for single sensory impairments. LPSS did not modify associations.DSI was associated with greater risk of having chronically high depressive symptoms and mild/moderate increasingly and chronically highly anxious symptoms. DSI was associated with an ~99 % increased risk of having chronically depressive symptoms group as well as with a ~60 % increased risk of being mild/moderate increasingly anxious and ~86 % increased risk of being chronically anxious. Interestingly, DSI contributed greater risk of having chronically high depressive symptoms and mild/moderate increasingly anxious symptoms than the individual sensory impairments. Low perceived social support does not appear to modify the association of DSI with the long-term depressive and anxiety symptom trajectory classes.	Although there is a high prevalence of DSI among older adults, it is poorly understood (Davila et al., 2009), under-recognized, and underdiagnosed (Heine and Browning, 2002). There is a lack of consensus as to what clinical cut points can be used to define HI and VI, and structural barriers may exist for those with HI and VI. Those with DSI may experience diminished quality of life (Brennan et al., 2006; Dalton et al., 2003). This feeling of diminished quality of life could be associated with the onset of depression and anxiety. By recognizing DSI as being part of the factors that influences depression and anxiety, this can lead to better rehabilitation options and increase communication-based situations (Heine and Browning, 2002). Rehabilitation can be employed for compensatory strategies, modifications in environment, and use of assistive tools on an as-needed basis.	
In fully adjusted models, those with dual sensory impairment had higher prevalence rates of limitations with mobility, self-care, and household activities compared to those with no sensory impairment and those with a single sensory impairment (Table 2). The prevalence rate of greater mobility limitations was 1.19 times (95% confidence interval [CI] = 1.10–1.30) higher among those with a single sensory impairment and 1.45 times (95% CI = 1.28–1.63) higher among those with dual sensory impairment relative to those without sensory impairment (p-trend< 0.001). The prevalence rate of greater limitations in household activities was 1.19 times (95% CI = 1.11–1.28) higher among those with a single sensory impairment and 1.54 times (95% CI = 1.37–1.72) higher among those with dual sensory impairment relative to those without sensory impairment (p-trend < 0.001). We also found that having dual sensory impairment (relative to no sensory impairment) was associated with greater difficulty with mobility and self-care activities to a similar extent as to having dementia (relative to not having dementia) (similar magnitude of PRRs). The association with limitations in [illegible]	NA	NA	NA	In a nationally representative sample of Medicare beneficiaries in the United States, we found that older adults with dual sensory impairment had greater limitations in mobility, household, and self-care activities compared with those without sensory impairment and those with a single sensory impairment, after adjusting for dementia, sociodemographic characteristics, and other functional and health status variables. Having both dual sensory impairment and dementia was associated with the highest levels of limitations in self-care and household activities.	Expanding Medicare coverage to include vision and hearing services could help optimize independence and functioning among older adults, including those with dementia. The regulation of over-the-counter hearing aids sales for the treatment of mild to moderate hearing loss under the Over-theCounter Hearing Aid Act of 2017 is another example of a policy that could have a positive impact on this issue. However, to date the Food and Drug Administration has not released regulations governing the samples of overthe-counter hearing aids	
NA	NA	NA	NA	In a nationally representative sample of Medicare beneficiaries, those with dual sensory impairment had significantly higher odds of dissatisfaction with perceived quality of care relative to those without sensory impairment in a model adjusted for sociodemographic and health covariates. In secondary analyses, sensory impairment was associated with higher odds of dissatisfaction with aspects of communication with patients and access to care relative to those without sensory impairment, including information provided about what was wrong and ease to get to a doctor. dual sensory impairment was associated with greater dissatisfaction with quality of care. Those with hearing or vision impairment alone were approximately 30% more likely to report dissatisfaction, although this finding was not statistically significant.	NA	To our knowledge, this is the first study demonstrating that those with dual sensory impairment had the greatest odds of dissatisfaction with ease of transportation to doctors among sensory groups relative to those without sensory impairment. People with vision impairment have identified physical access barriers and transportation needs as challenges to obtaining health car
Dual visual and hearing impairment was associated with higher odds of IADL decline compared to women with no sensory impairment (OR: 2.61, 95%CI: [1.50; 4.54])	NA	NA	NA	Results suggest that among autonomous older women, visual, and to a lesser extent, hearing impairment, have a short-term negative impact on their ability to perform daily activities, with some evidence of a multiplicative effect between sensory impairments. Among 4000+ French women aged 76 and older, all autonomous at baseline, those with a dual sensory impairment were at more than a 2.5-fold higher risk of IADL decline after a 4-year follow-up. Visual sensory impairment appeared to	Managing sensory impairments may be one of the early preventive measures which, while promoting quality of life, could limit functional decline, and prevent institutionalization	
In LMM analysis with CERAD TS as outcome variable, after follow-up, the overall CERAD TS score increased (β 0.65, 95% CI 0.40–0.91, p < 0.001). There was a statistically significant time interaction with DSI in all models (1, 2, and 3; model 1: β −0.8, 95% CI = −1.08 to −0.51, p < 0.001; model 2: β −0.87, 95% CI = −1.17 to −0.57, p < 0.001; model 3: β −0.86, 95% CI −1.16 to −0.56, p < 0.001), but not with SSI. These results imply that the neurocognitive scores of the DSI group significantly decreased during follow-up compared to the other groups. The results indicated a significant effect of DSI on dementia baseline prevalence, dementia incidence, and cognitive decline. But no significant effects of visual (OR 1.27, 95% CI 0.67–2.44; HR 1.23, 95% CI 0.66–2.30; β−0.12, 95% CI −0.41 to 0.16, p = 0.397) or of hearing impairment alone (OR 1.15, 95% CI 0.35–3.79; HR 0.93, 95% CI 0.26–3.30; β −0.38, 95% CI −1.01 to 0.26, p = 0.245) were observed. In the analysis, single disease (OR 0.97, 95% CI 0.71–1.32, p = 0.846) and dual disease (OR 0.75, 95% CI 0.31–1.60, p =0.487) did not have a significant effect on the prevalence of dementia. Dual disease (HR 1.95, 95% CI 1.05–3.64, p = [illegible]	NA	NA	NA	DSI was associated with baseline prevalence of dementia, incidence of dementia, and cognitive decline over the follow-up period, but the presence of visual impairment or of hearing impairment alone did not show such association. The results of the sensitivity analysis indicated that, even if there was no previous cognitive decline, DSI was associated with a future decline in cognitive function. visual only and hearing only impairment did not specifically influence dementia onset or cognitive decline, but there was a significant effect of DSI. In this study, differences in sensory deprivation, neurodegeneration, social isolation, and depression could explain why DSI had a significant effect but SSI did not. Sensory impairments might limit the neural resources needed for cognitive function by increasing the cognitive load.		This study showed that coexistence of visual and hearing impairments increases dementia prevalence, incidence, and cognitive decline but visual and hearing impairments alone do not.
Notably, participants with DSI showed the highest prevalence of cognitive impairment (50.2%), depression (53.1%), and anxiety (16.9%). Specifcally, HI (OR 2.05, 95% CI 1.75–2.40) and VI (OR 2.02, 95% CI 1.70–2.39) increased the prevalence of cognitive impairment compared with the participants with NSI. A similar result was found in the association between sensory impairment and depression and anxiety. However, the participants with VI showed a relatively higher prevalence of depression (OR 1.77, 95% CI 1.57–1.98, vs. OR 1.24, 95% CI 1.11–1.38) and anxiety symptoms (OR 1.66, 95% CI 1.40–1.96, vs. OR 1.51, 95% CI 1.28–1.78) compared to those with HI. Participants with DSI showed the highest prevalence of cognitive impairment (OR 4.19, 95% CI 3.58–4.91), depression (OR 1.94, 95% CI 1.71–2.20), and anxiety (OR 2.29, 95% CI 1.91–2.74)	Men had a significantly higher prevalence of hearing impairment than women (21.7% vs. 17.2%, P=0.006). However, men had a signifcantly lower prevalence of vision impairment (12.6% vs. 16.4%, P<0.001) than women. After stratifcation by gender, a statistically signifcant association was found in both men and women between sensory impairment (HI, VI, and DSI) and cognitive impairment, depression, and anxiety. Remarkably, the male participants with sensory impairment (HI, VI, and DSI) presented a higher prevalence of cognitive impairment, depression, and anxiety than the female.	NA	NA	Both single and combined sensory impairment associated with an increased prevalence of cognitive impairment, depression, and anxiety. Furthermore, DSI posed an additive impact on cognitive impairment, depression, and anxiety beyond that attributable to single sensory impairment (HI or VI). Subgroup analyses showed that the males showed a higher impact of sensory impairment on cognitive impairment, depression, and anxiety than the females.	Policy-makers should raise awareness about the risks of sensory impairment and provide health services to older adults sufering from sensory impairment.	

). Vision loss did not have a cross sectional relationship with depression symptom severity (Table 2). However, VL was associated with increased depression symptoms at 6 years in all models (b = 0.0220, SE = 0.01, P = .034). Hearing loss was associated with increased depressive symptoms at baseline in the fully adjusted model (b = 0.1750, SE = 0.07, P = .019), yet this relationship was not significant longitudinally. For DSL, there was no cross sectional relationship with depression; however, DSL was associated with increased depression symptomatology over time in all models (b = 0.0413, SE = 0.02, P = .007). Sensitivity analyses showed that further adjustment for social network and social activities did not attenuate the main depression results. All tested interactions were nonsignificant. Anxiety symptoms were also low in severity (median = 4, interquartile range = 4 5; HSCL range = 4 16). Vision loss was also not significantly related to anxiety symptoms, either at baseline or after 6 years (Table 3). Hearing loss was associated with increased anxiety symptoms at baseline, even after full adjustment for covariates (b = 0.1765, SE = 0.08, P = .026); [illegible]	Vision loss was more prevalent amongst women (58.6%, n = 318), whereas HL was less common among women (37.7%, n = 107).	NA	NA	Dual sensory loss was found to have a longitudinal relationship with depression, but not anxiety. Our analysis also demonstrated that VL, HL, and DSL have different mental health profiles, with HL related cross sectionally to both depression and anxiety, whereas VL and DSL have a longitudinal association with depression. Dual sensory loss poses an additive depression risk longitudinally beyond that attributable to VL.	This paper provides a strong rationale for improving health services and interventions to better meet the mental health needs of older adults with sensory loss(es). An increased focus on optimising aids and corrections is needed, given that use of assistive aids has been shown to reduce depression and mental health burden. This is particularly important given the high rate of correctable VL in older populations. Furthermore, older adults with VL and DSL, particularly, would benefit from depression intervention, while those with HL appear to have an immediate need for support regarding both depression and anxiety. Treatment of depression in those with VL is especially critical given the bidirectional nature of the VL depression relationship. Interventions might also aim to facilitate acceptance of the loss(es), which has been shown to lead to adjustment and reduced depression over time. It is particularly pertinent that services are directed towards older adults with sensory loss, given that over one third of older adults with VL and co morbid mental disorders do not receive mental health services.
NA	NA	NA	NA	The overall findings show that despite diagnosis with a DSL, the majority of older adults continue to use oral communication and report residual vision and/or hearing function. For those who used oral communication, the majority could participate in one-to-one and group conversation, which was not true for those who used manual. communication. No association between severity of DSL or vision loss was found in this group. Severity of hearing loss was associated only with ability to participate in group conversations and withdrawal from group conversation. Regardless of communication mode, the rate of report of wider participation difficulties was similar, as for withdrawal from conversation, loneliness, and social participation outside of the home	Firstly, there needs to be an awareness about the diverse communication modes and systems used by older adults with DSL. Secondly, the communication mode, needs, and preferences of the individual with DSL should be considered and assumptions about preferences should not be based on the severity of an individual's sensory losses. Finally, when assessing the communication skills of older adults with DSL, a full range of their communication possibilities should be considered, not only the ability to function using one modality. First, there needs to be conversation partners available who are able to communicate using your preferred mode/system or all communication needs to be mediated by an interpreter. Then, group conversations between sign language users with visual loss or users of tactile signed languages become practically difficult.<br>The implication of these findings is that whether communicating through an oral or manual mode, older adults with DSL require special consideration to support their participation in communicative activities.<br>These considerations could include strategies to modify the environment (e.g. reducing background noise and back lighting), the behaviours of communication partners (e.g. using slower, clearer speech and checking comprehension), and the individual's own communication style (e.g. communication repair strategies) to maximize communication participation
Clients with DSI were roughly twice as likely to have an association with impaired cognitive performance, as measured with the CPS (2.02; CI = 1.99, 2.05]), and were 30% more likely to be associated with a diagnosis of dementia other than Alzheimer's disease (1.29; CI = [1.27, 1.31]). They were no more likely to have a diagnosis of Alzheimer's disease (0.97; CI = [0.95, 1.00]) (Table 1). Experiencing DSI was associated with a greater risk of being diagnosed with multiple chronic health conditions and having impairments on both ADLs (1.69; CI = [1.66, 1.71]) and IADLs (2.04; CI = [2.01, 2.07]). The greatest proportions of clients with moderate/severe difficulty being understood had highly impaired hearing and highly impaired vision (62.6%), and moderate/severe difficulty understanding others was the most common in clients with highly impaired hearing and severely impaired vision (75.7%) (Table 2). Similarly, the same pattern was also true for communication decline, with the largest proportion (38.9%) found in clients with highly impaired hearing and severely impaired vision. The variable measuring selfreported loneliness did not show a [illegible]	NA	NA	NA	not only were home care clients with DSI worse off on measures of receptive and expressive communication, but they were also at greater risk for worsening of their communication ability over time. These same issues have been documented in community-dwelling older adults with DSI as communication requires a greater degree of concentration and breakdowns in communication can result in a fear of being offended or isolated, which can create an aversion to social contact and activities (Heine & Browning, 2004). The ability to effectively communicate and understand instructions from care providers and health professionals can be essential in meeting the needs of the client.	It is anticipated that these results will highlight the use of the RAI-HC as an important decision-support tool. The data collected from each client are used to individualize the care received, which becomes even more essential when we consider the individual level of hearing and vision impairment within DSI. By using the items, scales, and QIs within this population, we can draw attention to possible issues, and help the home care sector in their efforts to continually enhance the care that is being provided to their clients.
The hierarchical multiple linear regression model showed that depressive symptoms, loneliness and social support were all independently associated with mental HRQOL (see table 5). All relationships remained significant after controlling for confounders.	NA	NA	NA	Results from this study provide the first evidence that psychosocial well-being in Usher syndrome is associated with HRQOL in a UK population with Usher syndrome. This work adds to a growing area of research that is showing the importance of considering psychosocial well-being in people who have Usher syndrome	It is recommended that those professionals who work with this population should consider routinely screening for psychosocial well-being alongside monitoring physical health. It is also suggested that discussions around social support should also take place as higher levels of social support are linked with improved HRQOL. It will also be important that healthcare professionals be mindful of the importance of themselves as sources of social support for people with Usher syndrome and other sensory impairments. In order to ensure that they are supporting people to the best of their abilities they should make sure all support and screening for psychosocial well-being is accessible to people with hearing and visual impairments in line with Department of Health recommendations.
Among participants with dementia, those with DSI had higher odds of hospice use in most models (OR = 2.11; 95% CI = 1.05-4.21 for model 4). Similarly, DSI, but not VI or HI, was associated with hospice use in people without dementia. Total annual healthcare costs per participant were consistently higher among those with dementia compared with those without dementia across all models	NA	NA	NA	In a representative sample of Medicare beneficiaries, we found that the presence of self-reported sensory impairments was generally associated with increased healthcare utilization, including inpatient admissions, and total annual healthcare cost. Sensory impairments were common in this population, with 18% of those without dementia and 30% of those with dementia reporting combined VI and HI. We extend these findings by showing that DSI is a risk factor for hospitalization regardless of dementia status	the development of effective screening tools for cognitive impairment in this population will be crucial to identifying those at highest risk. While the Over-the-Counter Hearing Aid Act of 2017 is the first step toward ensuring access to affordable hearing aids, legislation that expands access to hearing rehabilitative services, such as communication counseling, is equally important. Future studies can help to determine if increasing coverage for restorative sensory care services will result in decreased overall healthcare cost. Additionally, focusing on sensory health within the context of preexisting dementia care programs may help to further promote functional independence and decrease healthcare spending. Another important consideration in this population is that hospitalization often represents a sentinel event that precipitates subsequent decline and institutionalization due to hospitalization-associated disability and delirium. System and physician-led interventions, such as promoting early mobility, reducing polypharmacy, and providing hearing aids and glasses in the hospital, may be opportunities to prevent this chain of adverse outcomes.

People with VI (OR = 1.760, 95%CI: 1.552–1.995) and HI (OR = 1.598, 95%CI: 1.315–1.943) have a higher chance of developing depression. People with DSL are 1.685 times more likely to be depressed than those who have no sensory loss (OR = 1.658, 95%CI:1.566–1.812). Participants who are female (OR = 1.696, 95%CI: 1.576–1.825), unmarried (OR = 1.390, 95%CI: 1.242–1.557) and lives in the rural areas (OR = 1.281, 95%CI:1.175–1.395) have higher risk of developing depression symptom.	It shows that DSL, gender, age, education, marriage status and residence were significantly related to the depression symptoms (P < 0.05). Among the significant variables, sensory loss, female, unmarried and living in rural areas are risk factors of depression.	NA	NA	The results show that the DSL is associated with a higher healthcare expenditure and the higher chance of the incidence of CHE. Furthermore, our study fully supported that depression has a significant mediating effect in the relationship of DSL and healthcare costs and the incidence of CHE. More specifically, the results show that DSL is associated with a higher likelihood of depression, which ultimately leads to an increased healthcare costs and CHE. Gender, age, residence, marriage, public insurance and consumption ability are related with increased healthcare expenditures and CHE.	Considering that DSL, either by itself or in the association with depression, can lead to excessive consumption of healthcare resources and result in financial catastrophic for both families and social financing system, concerted efforts should be made to prevent economic adversities due to DSL. First, healthcare professionals and policy-makers shall raise awareness of the potentially more cost-effective investment in early-stage interventions to prevent or slow down the occurrence of DSL and depression. The development of effective screening tools for DSL and depression, as well as the hierarchical management of this population, will be crucial (Deardorff et al., 2019; Ding et al., 2022). Second, rehabilitative or restorative treatments focused on DSL, such as hearing aids or cataract surgery, may help control excess healthcare costs. Legislation that expands access to DSL rehabilitative services, including communication counseling, should also be seriously considered (Reed et al., 2018). Additionally, early interventions for depression associated with DSL, such as psychological counseling, may further promote control of healthcare expenditures. Besides, our findings suggest that there is an association between DSL, depression and healthcare costs. The complex mechanism of DSL on healthcare costs emphasizes the overwhelming necessity of redesigning health services and developing alternative interventions with a more holistic approach tailored to the complex needs of individuals	
Compared with the unimpaired group, after adjustment for sex and age, participants with HI were at higher risk of CVD-related mortality [HR: 1.52 (95% CI: 1.17–1.97)], whereas participants with DSI were at higher risk of death from any cause [HR: 1.50 (95% CI: 1.19–1.90)] or from CVD [HR: 1.80 (95% CI: 1.24–2.61)], as shown in Table 2. After adjusting for established mortality risk factors, including smoking, BMI, hypertension, diabetes, self-reported health status, cognitive status, self-reported history of falls, total cholesterol, baseline CVD history and hearing aid use, DSI remained associated with all-cause mortality [HR: 1.43 (95% CI: 1.11–1.85)] and HI and DSI remained associated with CVD mortality [HRs: 1.70 (95% CI: 1.27–2.27) and 1.78 (95% CI: 1.18–2.69), respectively].	Among those with a given impairment, women were more likely than men to have VI (10.5 versus 7.6%, P = 0.10), whereas men were significantly more likely than women to have HI or DSI (29.9 versus 22.0%, P < 0.01 and 8.6 versus 5.7%, P < 0.01, respectively). After adjusting for age and sex, the impaired groups had significantly less education, poorer self-reported health, more depressive symptomology, cognitive impairment, walking disability and higher rates of diabetes. Men were more impaired and more severely impaired in vision and hearing compared with women.	NA	NA	This population-based study of community-dwelling older people found sensory impairment to be common and, for men with HI or DSI, associated with all-cause and CVD-related mortality. Whether sensory deficits in men are an indicator of ageing or frailty, physical manifestations resulting in reduced social competence or a reflection of other adverse health states is unclear	Regular assessment of sensory impairment and rehabilitation services targeted for those with decrements in hearing and vision in old age can promote enhanced quality of life, health and longevity	
the odds of cognitive impairment by sensory loss among those aged 65 and older. For both sexes aged 65 and older, in comparison to those with no sensory loss, there was a doseresponse in the odds of cognitive impairment among those with hearing impairment only (OR = 2.66; 95% CI = 2.64, 2.68) and vision impairment only (OR = 3.63; 95% CI = 3.59, 3.67) having markedly lower odds than those with dual sensory impairment (OR = 8.16; 95% CI = 8.07, 8.25). Similar findings were evident for both men (hearing impairment only: OR = 2.77; 95% CI = 2.74, 2.80; vision impairment only: OR = 3.89; 95% CI = 3.81, 3.96; dual sensory impairment: OR = 9.02; 95% CI = 8.88, 9.17) and women (hearing impairment only: OR = 2.58; 95% CI = 2.55, 2.61; vision impairment only: OR = 3.50; 95% CI = 3.45, 3.55; dual sensory impairment: OR = 7.55; 95% CI = 7.44, 7.66). Table 3 also examines both sexes combined by 10-year age cohorts. The magnitude of the odds of cognitive impairment by sensory impairment was greatest for the [illegible]	However, men were overrepresented in the hearing impairment only category (56.9% male versus 43.1% female)	NA	NA	The results of this nationally representative study of 5.4 million American older adults support an independent association between cognitive impairment and hearing impairment and vision impairment alone, as well as dual sensory impairment. One in six older adults with cognitive impairments (16.0%) had dual sensory loss compared to only one in 53 (1.9%) of their peers without cognitive impairment. When compared to older adults aged 65 and older without any sensory impairment, older adults with hearing impairment only or vision impairment only had 2.66 and 3.63 higher odds of cognitive impairment, respectively. Among older adults with dual sensory impairment, the odds of cognitive impairment increased eight-fold. For older men specifically, those with hearing impairment only or vision impairment only had 2.77 and 3.89 higher odds of cognitive impairment, respectively. Among older women, those with hearing impairment only or vision impairment only had 2.58 and 3.50 higher odds of cognitive impairment, respectively. The odds of cognitive impairment increased nine-fold among men and more than seven-fold among women among those with dual sensory impairment.	It is essential that practitioners and researchers consider the full impact of sensory impairment on cognitive testing methods, as both auditory and visual testing methods may fail to take hearing and vision impairment into account . When performing cognitive tests on older adults with sensory impairments, practitioners should ensure they are communicating audibly and/or using visual speech cues for hearing impaired individuals, eliminating items from cognitive tests that rely on vision for those who are visually impaired, and using physical cues for individuals with hearing or dual sensory impairment, as this can help increase the accuracy of testing and prevent confounding	
older adults with hearing loss had a significantly faster rate of cognitive decline as they grew older. Compared with those with normal hearing, older adults with hearing loss declined 0.16 more points in cognitive function as they grew 1-year older ($\beta = -0.16$, $P < .05$). Older adults with vision loss did not have a significantly faster rate of cognitive decline. Compared with those with normal vision older adults with vision loss declined 0.06 more points in cognitive function but not significant ($\beta = -0.06$, $P = .41$). Compared with those with no sensory loss, older adults with dual sensory loss declined 0.23 more points in cognitive function as they grew 1-year older ($\beta = -0.23$, $P < .05$). Such decline was larger than that of hearing loss alone ($\beta = -0.16$, $P < .05$), suggesting a potential additive risk effect of dual sensory loss on cognitive decline. When sensory loss was defined as either visual or hearing loss, older adults with only one type of sensory loss declined 0.06 [illegible]	NA	NA	NA	We found that objectively measured hearing loss was associated with a faster rate of cognitive decline over an 8-year follow-up period. Older adults with dual sensory loss also had a significantly faster rate of cognitive decline than older adults with normal sensory function, and this effect was modestly greater than for hearing loss alone. These associations remained significant after adjusting for demographics, socioeconomic status, health status, and lifestyle factors. However, no significant associations were found between vision loss and cognitive decline. Our study found that older adults with dual sensory loss had a faster rate of cognitive decline than those with no sensory loss.		
NA	NA	NA	NA	We show that older adults with moderate to severe hearing impairment compared to those with normal hearing had a greater likelihood of being retired at baseline examination. Further, those who used a hearing aid reported a significantly lower adjusted mean retirement age (1-year). Older adults who had concurrent presenting visual impairment and moderate to severe hearing loss compared to those with no impairment had lower adjusted mean retirement age. Hearing loss at baseline showed a marginally non-significant association with the decision to retire by the 10-year follow-up. Study participants with moderate to severe hearing loss (> 40 dB HL) versus those with normal hearing not only had a higher likelihood of reporting retirement, but also retired around one to two years earlier.		
NA	NA	NA	NA	Cross-sectional analyses demonstrated that older adults adhering to established dietary guidelines compared to those who did not were less likely to have DSI. This observed association was independent of potential confounders such as education level, smoking and type 2 diabetes. However, this association did not persist over the 5-year follow-up period.	Moreover, the follow-up period of 5 years is short and the mean age of the cohort is relatively old, hence, most of the influences from total diet could have already been exerted, resulting in the significant associations association being observed with prevalent DSI, but not with incident of DSI. hence, future prospective studies with longer follow-up and larger sample size are likely to provide more precise estimates of risk reduction that can be achieved with a healthy dietary pattern among older adults.	NA
A minority of individuals in the sample had depressive symptoms (14.2%), less than half showed signs of cognitive impairment (41.1%), and 16.2% had DSI.	NA	NA	NA	The overall prevalence rate of symptoms of depression was 14% in this large sample of home care recipients in Ontario. This rate is consistent with previous research,1,5 and is similar to other home care research using the RAI-HC. The prevalence of DSI was 16%. This is higher than most studies examining older adults, which could be due to the nature of the way in which DSI was defined. The most important risk factors for predicting symptoms of depression were poor self-reported health, cognitive impairment and speaking a primary language other than English or French.	Future research following clients over time, who are re-assessed with the RAI-HC, can also shed light on the important causal pathways linking factors, such as self-reported health, DSI, primary language, and cognitive impairment with the risk of depression in the home care population.	NA

However, those with DSI were much more lik	NA	NA	NA	Our results showed that those with DSI had higher rates of cognitive im	Research on older adults with DSI lags behind that helping us understand other complex health issues experienced in this population. More studies are needed, ideally at the national level, to understand more fully the prevalence of DSI and the needs and abilities of persons with this impairment. Organisations that work with older adults need to gather information about vision and hearing impairments since the majority of those with DSI are at least 65. Through better awareness of this disability, and by creating new policies and services to support these individuals and their families, there is tremendous potential to improve their capacity to be independent and to optimize quality of life.	NA
Differences between the groups exist for nearly all variables (except for the number of important people in regular contact). Moreover, compared to individuals with no sensory impairments, individuals with DSI consistently reported worse psychosocial factors. For example, while the average depressive symptoms score among individuals without sensory impairment was 5.7 (SD: 5.5), it was 7.1 (SD: 6.0) among individuals with either visual or hearing impairment, and 7.5 (SD: 5.9) among individuals with DSI.	NA	NA	NA	Individuals with DSI had higher rates of specialist healthcare v	The study suggests that efforts to prevent or delay DSI could reduce the economic burden on healthcare systems. This is due to the associated higher healthcare use among individuals with DSI.	NA
One way ANOVA revealed significant between group differences in cognitive function (P < .05) and quality of life (P < .05). For the cognitive score, the no impairment group had the highest mean score, followed by vision loss only, hearing loss only, and DSI groups. Bonferroni post hoc analysis revealed that DSI and hearing loss only groups had significantly lower scores than the no impairment group (P < .05).	NA	NA	NA	DSI significantly affects the quality of life and cognitive function in older adults. Sociodemographic factors like education play a critical role in mediating these effects. DSI and hearing loss alone are associated with lower cognitive performance.	Future studies using a larger number of participants are warranted. Longitudinal studies are warranted to explore the causal relationship between sensory impairment and mental health.	NA
DSL, poor vision only or poor hearing only all had no significant influence on ageing in place but all had a significant influence on depression;	NA	NA	NA	Dual sensory loss in older women is linked to worse general health, higher depression rates, and decreased social activity. The prevalence and impact of DSL increase with age, particularly affecting women living alone	Early identification of DSL is essential to minimize its effects and ensure continued well-being. Targeted interventions for older women with DSL are recommended to address their specific challenges.	NA
NA	NA	NA	NA	Significant disparities exist in the prevalence of sensory impairments based on socioeconomic factors, with poorer vision and hearing associated with lower SES and rural residency. The study highlights a large unmet need for sensory aids among those with impairments, as many do not use glasses or hearing aids despite experiencing sensory losses.	We recommend that further training in sensory loss and rehabilitation be made available to primary care health professionals, particularly those who have high older patient caseloads. There is also a need for the training of hearing specialists in hearing care. Some multinational private hearing care companies are now setting up hearing training centers in China but public facilities are limited. Finally, we recommend that consideration be given to the inclusion of greater screening and sensory aid reimbursements in medical insurance schemes in China.	NA
Respondents with DSL had a higher proportion of reporting depression (18.1%), life dissatisfaction (14.58%), any chronic disease (76.72%) and difficulty in completing ADLs (11.21%) and IADLs (23.28%), compared to the group with no DSL. In addition, there are significant comorbidities between DSL and chronic diseases, ADLs or IADLs. About 44.95, 6.77, and 13.03% had DSL together with any chronic disease, ADL or IADL, respectively. DSL had significant influences on both mental health and wellbeing, however, the absolute value of association between DSL and depression (0.785) in Model 1 is higher than that of the association between DSL and life satisfaction (−0.593) in Model 3. (2) Older people who have any chronic diseases are more likely to report depression but there is no significant difference in their reporting of [illegible]	NA	NA	NA	DSL was significantly associated with depression and reduced life satisfaction among older adults. It also correlated with difficulty in Activities of Daily Living (ADL) and Instrumental Activities of Daily Living (IADL), indicating a broad impact on daily functioning and mental health.	It is recommended that health services in China screen for DSL in older adults and develop integrated services to assist with the management and rehabilitation focusing on both functional and mental health issues.	NA
Both VI and HI were significantly associated with depressive symptoms in three waves, even after adjusting for other variables, including demographic variables, health-related variables, and social isolation. As shown in Table 3, we detected an additive interaction between VI and HI on depressive symptoms in Model 1 (RERI [95 % CI]: 32.27 [25.01 to 39.54] in wave 1, 30.55 [22.34 to 38.76] in wave 2, and 149.17 [114.90 to 183.46] in wave 3]).	NA	NA	NA	Vision and hearing impairments were significantly associated with increased depressive symptoms over time. This association was consistent even after adjusting for various covariates, including social isolation.	These findings imply that early correction and treatment for VI and HI could effectively prevent depressive symptoms among older adults in China. Furthermore, incorporating mental health screening for older adults with VI and HI as a part of geriatric care could be beneficial. Future studies are recommended to focus on investigating the underlying mechanism by which VI and HI influence depressive symptoms.	NA
Among 1352 participants who had MMSE assessed at both the 5- and 10-year follow-up examinations, possible cognitive decline was found in 9.5% (9), 11.1% (24) and 18.8% (6) of persons with VI, HL and DSI, respectively, compared to 7.6% of controls over the same 5-year period.	NA	NA	NA	The presence of visual impairment, hearing loss, or dual sensory impairment was not independently associated with a subsequent decline in cognition over 5 or 10 years after adjusting for age and sex.	Longitudinal studies with larger sample sizes, clearly defined sensory impairment duration, and the use of appropriate testing tools to disentangle cognitive impairment from sensory impairments, are needed to confirm or refute the hypothesis that sensory impairment may lead to an increased risk of cognitive decline among older persons.	NA
NA	NA	NA	NA	he study found no significant associations between the severity of sensory loss and relational strain in terms of excessive demands, worries, or conflicts with close social relations. However, those with total blindness and profound deafness had higher odds of experiencing conflicts with their children and personal support workers compared to those with residual vision and hearing.	We recommend that future studies look further into the nature of the relational strain experienced by people with DSL and investigate how other factors such as personal characteristics, coping, and communication abilities and support might influence their social relations.	NA
A significant association was found between thoughts of SH or suicide and scores on the MDI, with participants who reported more symptoms of depression also being significantly more likely to report having thoughts of SH or suicide (OR ¼ 1.19, 95% CI [1.13, 1.25]).	NA	NA	NA	17% of participants reported thoughts of self-harm or suicide in the past two weeks. Factors such as non-participation in social activities, poorer self-rated health, and presence of more depressive symptoms were associated with these thoughts.	This finding highlights for professionals the importance of ongoing mental health screening and support for older adults with DSL, especially in ensuring that mental health services are accessible for this group in terms of both mobility and communication. It also highlights that professionals should be alert to the risk of SH/SI in this group, as these behaviors are often not considered in screenings of depressive symptoms. Further, the possibility that increased social participation and emotional support may act as protective factors for older adults at risk of SH/SI should be considered.	NA
During the mean follow-up period of 11.1 years (IQR: 10.9–11.4 years), a total of 1,135 (1.00%) participants developed all-cause dementia. Of these incident cases, 789 were in the NSI group, 42 in the VI-only group, 283 in the HI-only group, and 21 in the DSI group	NA	NA	NA	Visual, hearing, and dual sensory impairments were significantly associated with an increased risk of developing dementia. DSI was particularly associated with a higher risk of incident dementia.	Preventative or therapeutic interventions for VI and HI should be made widely available and their significance beyond the scope of improving sensory functions should be highlighted.	NA
Over 11,392 person-years of follow-up, 321 participants (15.6%) developed all-cause dementia: 14.3% in participants with no sensory impairments, 16.9% in those with one sensory impairment, and 28.8% in those with DSI.	NA	NA	NA	Dual sensory impairment was significantly associated with a higher risk of all-cause dementia and Alzheimer's disease. The hazard ratio for all-cause dementia was 1.86, and for Alzheimer's disease, it was 2.12.	DSI may serve to identify older adults at high risk of dementia. Future studies should characterize the exact role of sensory impairments and identify whether treatments that improve sensory function can modify this risk. Because the public health burden of dementia will increase over the next three decades, evaluation of vision and hearing function in older adults may help identify patients at elevated risk of developing dementia.	NA

Over 14 455 person-years of follow-up, 307 participants (10.5%) developed dementia. Compared with no sensory impairment, DSI was associated with increased risk of all-cause dementia (hazard ratio [HR], 2.60; 95%CI, 1.66-2.06; P < .001), AD (HR, 3.67; 95%CI, 2.04-6.60; P < .001) but not VaD (HR, 2.03; 95%CI, 1.00-4.09; P = .05).	NA	NA	NA	DSI was associated with a significantly increased risk of all-cause dementia and Alzheimer's disease but not vascular dementia. The hazard ratios were notably high, indicating a strong association between DSI and increased dementia risk.	Additional studies are needed to understand whether sensory impairments are a causal risk factor for dementia or a marker of incipient dementia. With the public health burden of dementia expected to increase in the coming decades, our findings suggest that evaluation of vision and hearing should play an important role in preventive strategies for dementia.	NA
In models adjusted for informant dyadic relationship and socio-demographics, both lower visual acuity (B = 0.23, SE = 0.10) and self-reported hearing difficulty (B = 0.15, SE = 0.06) were associated with increased levels of distress. The increased informant distress associated with poor visual acuity was attenuated after adjusting for neurocognitive disorder and health conditions (p = 0.069). A significant interaction between vision and hearing remained after multivariable adjustment ($\chi 2(1) = 6.73$, p = 0.010)				Both poor visual acuity and self-reported hearing difficulty were associated with increased levels of distress in informants. An interaction effect showed that the combination of both impaired vision and hearing led to higher levels of distress among informants compared to only one sensory impairment or none.	These findings highlight the need for better assessment and treatment of sensory disability, including advice for family and informal caregivers on strategies to manage communication difficulties, mobility limitations, and other challenges arising from sensory loss. Future research should investigate interventions to aid and support communication and other daily needs of older adults living with sensory impairment.	NA
Hazard ratios for depressive symptoms in those with visual, hearing, and dual sensory difficulties were 1.25 (95% CI 1.00–1.56, p = 0.047), 0.98 (95% CI 0.82–1.18, p = 0.82), and 1.67 (95% CI 1.29–2.16, p < 0.001), respectively, relative to those without sensory difficulty.				The study found significant associations between self-reported visual and dual sensory difficulties and the incidence of clinically significant depressive symptoms. No such association was found with hearing difficulties alone.	The current study provides a deeper understanding of the longitudinal associations between vision, hearing, and mental health that may help to inform public health interventions and future interventional research aimed at determining the role of maintaining late-life sensory health for promoting mental health. Such insights are vital to address the needs of a growing population of older adults in the United States and globally.	NA
During the mean follow-up period of 22.6 months, we identified AD onset in 1,194 (8.4%) participants. Of these incident cases, 870 were in the NSI group, 222 in the VI only group, 64 in the HI only group, and 38 in the DSI group. The log-rank test demonstrated a significant difference in AD incidence among the four groups (p < 0.001). The sex/age-adjusted model estimated the HR for developing AD (reference: NSI) to be 1.1 (95% CI: 1.0–1.3, p = 0.08) for participants with VI only, 1.4 (95% CI: 1.1–1.8, p = 0.011) for participants with HI only, and 1.7 (95% CI: 1.3–2.4, p < 0.001) for participants with DSI. The multivariable model estimated the HR for developing AD (reference: NSI) to be 1.0 (95% CI: 0.9–1.2, p = 0.60) for participants with VI only, 1.2 (95% CI: 0.9–1.5, p = 0.21) for participants with HI only, and 1.6 (95% CI: 1.1–2.2, p = 0.008) for participants with DSI.				Dual sensory impairment (DSI) conferred a significantly higher risk (HR: 1.6, CI: 1.1–2.2, p = 0.008) of Alzheimer's disease onset compared to those with neither sensory impairment (NSI). Visual and hearing impairments alone were not associated with incident Alzheimer's disease.	The prevention and treatment of sensory impairment may have clinical implications beyond the preservation of visual and auditory function. Increasing the availability and accessibility of such interventions for at-risk persons may not only improve their functional outcomes and quality of life, but could also help to reduce their future risk of AD.	NA
In the comparison of physical function in each group according to sensory impairment, muscle strength (handgrip strength) was statistically significantly lower in the VI only (24.1 7.5 kg) and DSI (22.8 6.9 kg) groups than in the NS group (25.8 7.6 kg) (p < 0.001). Regarding physical performance, the DSI group showed the poorest TUG test performance compared with the other groups (NS vs. DSI, 10.0 2.3 s vs. 11.9 3.5 s, p < 0.001). Statistically, the DSI group showed the lowest values of gait speed (NS vs. DSI: 1.1 0.2 m/s vs. 1.0 0.3 m/s, p < 0.001), CS-5 (NS vs. DSI: 11.1 3.4 s vs. 13.1 5.2 s, p < 0.001), and composite SPPB score (NS vs. HI only vs. VI only vs. DSI: 11.0 1.3 vs. 10.8 1.5 vs. 10.7 1.4 vs. 10.1 1.9 points; p < 0.001)				DSI was associated with higher odds of having lower muscle strength and poorer physical performance compared to single sensory impairments. Longitudinally, DSI at baseline increased the risk of deteriorating physical performance during the follow-up period more significantly than other forms of sensory impairment.	DSI increases the risk of deteriorating physical function (muscle strength and physical performance) to a greater extent than SSI in community-dwelling older adults. Therefore, more attention should be paid to the diagnostic evaluation and management of sensory impairment aimed at slowing the rate of decline in physical function in older adults.	NA
NA	NA	NA	NA	The crude prevalence of DSI among adults 50 years and older was 7.54%. There was a significant variance in DSI prevalence based on region, age, and socioeconomic factors. Higher social isolation and poor self-rated health status were strongly associated with DSI.	There are important differences in terms of prevalence of DSI in Europe, depending on socioeconomic and medical factors. Prevention of DSI does represent an important challenge for maintaining quality of life in elderly population.	NA
In comparison with participants without VL, HL, o	NA	NA	NA	This study revealed bidirectional associations between VL, HL, or DSL and depressive symptoms based on a nationally representative dataset of middle-aged and older Chinese adults. The results of this study indicated that the baseline status of vision, hearing, and dual sensory was independently associated with the risk of developing depressive symptoms, and the baseline status of depression was also independently associated with the risk of developing VL, HL, and DSL.	The mental health of people with VL and HL, especially those with DSL, should be focused on. Regular assessments of vision and hearing should be provided to people with depressive symptoms to ensure that interventions are provided at an appropriate time.	In the middle-aged and older Chinese population, people with VL, HL, and DSL are likely to develop depressive symptoms, and people with depressive symptoms are likely to report VL, HL, and DSL in the future.
After adjusting the covariates including age, gender, marital status, smoking, drinking, physical exercise, SRL, SRH and SRE, although the coefficient reduced, the groups with DSI (B = −6.302, 95%CI: −6.585, −6.020) was still significantly correlated with cognitive function. In addition, age (B = −0.134, 95%CI: −0.143, −0.126), gender (B = −1.174, 95%CI: −1.338, −1.011), SRL (B = −0.439, 95%CI: −0.614, −0.263), SRH (B = −0.485, 95% CI: −0.645, −0.325), SRE (B = −0.473, 95%CI: −0.657, −0.288) were negatively associated with anxiety. And respondents who were married (B = 0.281, 95%CI: 0.106, 0.455) or had the habit of physical exercise (B = 0.463, 95%CI: 0.305, 0.622) had a lower risk of cognitive impairment. In conclusion, the results of Model 1 and Model 2 suggested that sensory impairment was a risk factor for cognitive decline in older adults. As for the indirect pathways, the effect of DSI on cognitive function was significantly mediated by anxiety, with the effect value of −0.057. The independent mediating effect of depressive symptoms on the relationship between DSI and cognitive function was also significant, with the effect value [illegible]	NA	NA	NA	Sensory impairment and poor cognitive function are common experiences of older adults worldwide and are major contributors to the burden of nonfatal disease. There were some crucial findings as follows: Firstly, sensory impairment had a significant association with higher risk or prevalence of anxiety and depressive symptoms and poorer cognitive function. Secondly, anxiety and depressive symptoms also played partial and chain mediating effects on the association between sensory impairment and cognitive function. These associations remained significant after adjusting for the covariates.	Given that anxiety and depressive symptoms represent the most common mental disorders of later life, it would appear important to explore their contribution to negative outcomes such as poor cognitive function, in the context of sensory impairment. To better understand the possible relationship between sensory impairment, anxiety, depressive symptoms and cognitive function, clinical diagnosis results should be used in conjunction with more sophisticated neuropsychological tests.	NA
Three main results were obtained from this study by using representative national data. First, both self-reported VI and DSI were associated with more severe levels of depressive symptoms after 3 years of follow-up; however, the longitudinal association between HI only and depressive symptoms was nonsignificant. Second, DSI led to a higher OR for depressive symptoms than VI or HI only. Finally, the improvement in vision and/or hearing was still associated with an increased risk of depressive symptoms. These findings suggest that VI and DSI should be emphasized in future health intervention programs to reduce depressive symptoms.	NA	NA	NA	Our research also has some innovative findings. Compared to new-onset DSI, persistent DSI resulted in a higher risk ratio for depressive symptoms. The increase in depressive symptoms over time may be associated with further deterioration of HI and VI. Additionally, people with sensory impairment were more likely to experience depressive symptoms even after experiencing improvements in their vision and hearing status than those who self-reported that their vision and hearing remained good. The following may be reasons for this phenomenon. First, although the sensory function has improved, it may not have met the expectations of the respondents.	Future studies could consider using objective measurement approaches to compensate for the limitations of subjective reporting. Second, the practice of wearing glasses and hearing aids also causes a series of psychological and behavioral inconveniences, such as the discomfort of wearing assistive devices and the background noise of hearing aids (McCormack & Fortnum, 2013). Finally, we lack a long-term follow-up of the levels of depressive symptoms in respondents who self-reported improvements in vision or hearing status. Therefore, further studies are required to gain a deeper understanding of the relationship between improvements in vision and hearing status and long-term depressive symptoms.	NA

NA	NA	NA	NA	Several of the participants described the importance of rehabilitation services focusing on compensating for loss in function because these rehabilitative interventions mainly simplified for them in their daily life. Thus, they could continue with meaningful activities despite their health condition of DSL. Therefore, such rehabilitation interventions can be interpreted as related to the key terms of functional ability and intrinsic capacity, which are both components of healthy ageing (WHO, 2015). Many of the participants had positive experiences from rehabilitation interventions provided in groups with other older adults with similar health conditions. They expressed the importance of meeting with others, with whom they could exchange experiences and develop strategies for mastering their situation and acquiring skills	To gain equal access to health care for older adults, it is important to ensure that environments are accessible to meet their needs (WHO, 2015). For the particular population of this study, it is also important to adapt the environment to the DSL. DSL-friendly environments should include good contrasts, reduced glare, tactile markups, adapted sound and lighting, loop system and to the possibility for personal contact with the person in the clinic reception area (c.f WHO, 2015). The goal of rehabilitation is to promote participation such that the individual is able to continue with life-roles and activities in daily life that the person value (Saunders & Echt, 2012). When the rehabilitation services manage to fulfill these goals, the results indicate that the process of healthy ageing simultaneously seems to increase for older adults with DSL.	NA
The association between hearing impairment and cognitive impairment became not significant only among men. When health factors were added in Model 3, the association between vision impairment and cognitive impairment became not significant for both genders. In the final model, having hearing impairment and dual sensory impairment were significantly associated with cognitive impairment only among women. In terms of gender differences, the association between sensory impairment and cognitive impairment was not statistically significant.	The odds of having depression was 49% higher for women with vision impairment than women with no sensory impairments, while there was no significant difference in the odds of having depression between men with vision impairment and men without any sensory impairment. However, men with dual sensory impairment were more likely to be depressed than men having no sensory impairments, while no significant difference was found between women with dual sensory impairment and women without any sensory impairment. In terms of gender differences, the association between sensory impairment and depression was not statistically significant.	NA	NA	Corresponding to previous studies, sensory impairments were significantly associated with both depression and cognitive impairment though no statistical gender differences were found in above associations, gender-specific associations were found. For example, vision impairment was significantly associated with depression, and hearing impairment was significantly associated with cognitive impairment only among women. In addition, having dual sensory impairment was significantly associated with depression only among men, while having dual sensory impairment was associated with cognitive impairment only among women. The results of this study also suggest that hearing impairment is associated with cognitive impairment only among women. This study also suggests that there is a significant association between dual sensory impairment and geriatric mental health outcomes. Interesting finding is that dual sensory impairment was not significantly associated with depression in female population. This finding is similar to previous finding with Chinese older adults which did not differentiate gender.	In the future, it would be valuable to do a prospective study examining longitudinal change in geriatric mental health associated with sensory impairment among older Koreans.	NA
In multiple logistic regression analyses, depression was approximately fve times higher among the participants with DSI (OR: 4.90; 95% CI: 2.63–9.14) afer adjusting for other covariates	NA	NA	NA	One out of every four elderly in residential care in Hyderabad, India had depression. The elderly with DSI were fve times more at risk for depression. Also, depression was higher among those with single sensory impairment than in those without any sensory impairment. Several factors may have contributed to their depression. As vision and hearing are vital senses that one heavily depends on and is habituated to using routinely, their lack may be perceived as a grievous loss. Tus, one's inability to see clearly and or hear properly may predispose the elderly to depression. As the elderly in residential care are relatively more dependent on others for their daily living as compared to persons living at home with their family, it is likely that the efect of DSI can compound their difculties and worsen the level of depression compared to those who live with their family in the community setting. No comparative study has reported this in India.	In the current scenario, assessments and services are provided by diferent specialists. We need to adopt a holistic approach to jointly address the multi-morbidity associated with DSI and depression.	In conclusion, depression and DSI are common in the elderly living in residential care. Since patients with DSI are at higher risk of developing depression, assessment of depression should be performed as part of the routine practice in eye and ear examination clinics in India. A multi-pronged approach to address this important multi-morbidity is critical to improve the quality of life and facilitate healthy aging for elderly in India
The Kaplan–Meier survival analysis indicated that HI (log-rank $\chi 2$ = 10.42; P < 0.001) and DSI (log-rank $\chi 2$ = 39.92; P < 0.001) were linked to significantly higher cumulative dementia incidence compared to NSI. DSI had a significantly higher cumulative dementia incidence than HI (log-rank $\chi 2$ = 11.37; P = 0.001).	NA	NA	NA	The presence of a sensory impairment among older adults accessing LTCI increases their risk of developing dementia. Among our sample, 55.7% of older adults had either VI, HI, or DSI; more individuals had HI compared to VI. Among sensory impairments, DSI was the greatest risk factor for the development of dementia.	As our data were obtained from data of older adults accessing LTCI, living in a single city, generalisability of the sample is limited. Replication studies in other cities or prefectures are needed to confirm our results. Future studies are needed to develop appropriate interventions for sensory impairments with the overarching aim of preventing deterioration in care-need levels.	Our results showed DSI to be the greatest risk factor for the development of dementia. Furthermore, it was suggested that VI in older adults may increase sleep and circadian rhythm disorders, even in mild impairment. While dementia and BPSD are related to a deterioration in care-need level, previous studies have shown that interventions for sensory impairments may have a positive influence on health-related outcomes.
NA	NA	NA	NA	Vision loss (in men) and dual sensory loss (in 65- to 85-year-olds) were independently associated with low social network diversity. Vision loss and dual sensory loss (in 65- to 85-year-olds) were independently associated with low social participation. Hearing, vision, and dual sensory losses were each independently associated with loneliness and reduced availability of social support, respectively.	Further research is needed to determine mechanisms underlying the associations, and whether addressing sensory impairment yields benefits outside of simply improving sensory function.	
The prevalence of cognitive impairment was higher in individuals with dual sensory impairment (1.965, 95 % CI 1.46–2.65), followed by those with hearing impairment (1.473, 95 % CI 1.13–1.92) and vision impairment (1.457, 95 % CI 1.07–1.98). Multivariate analysis showed that the prevalence of cognitive impairment in the 75–89 age group was significantly higher among individuals with dual sensory impairment (1.916, 95 % CI 1.339–2.742), followed by those with hearing impairment (1.584, 95 % CI 1.15–2.18) and vision impairment (1.513, 95 % CI 1.04–2.21). Multivariate analysis showed that HR of mortality was 1.290 (95 % CI 1.01–1.65) in individuals with dual sensory impairment and cognitive impairment, and 1.225 (95 % CI 0.96–1.56) in those with hearing impairment and cognitive impairment relative to normal sensory and cognitive functions.	NA	NA	NA	Dual sensory impairment was the greatest risk factor for cognitive impairment, and those with dual sensory impairment and cognitive impairment had increased risk of mortality. The elders with dual sensory impairment and cognitive impairment had increased mortality in our study.	Healthcare providers should be aware of the increased risk of cognitive impairment and mortality in those with sensory impairment. Further studies are needed whether early identification and management of sensory impairment could improve cognitive outcomes and survival in elders.	NA
NA	NA	NA	NA	Looking at the individual trajectories of IADL items, household tasks, traveling, grocery shopping, preparing a meal, and walking were activities with the most rapid decline, with household tasks having the highest probability of decline in the younger-old with VL (0.18) and DSL (0.19). Trajectories depict a decline in IADL competence with age. Household tasks, traveling, grocery shopping, preparing a meal, and walking showed the most rapid decline	Future longitudinal research should include severity of SL, technology use as well as context and coping strategies and IADL capacity.	NA
NA	NA	NA	NA	The findings suggest that the DSI older adults feel threatened in their existence as a social human being; they feel unable to reach out to others, to be aware of what is happening in their environment, or to discuss and negotiate aboutthe care they receive. In addition, they feel hindered in using their physical belongings properly, and in controlling the professionals' support and handling of their belongings. Our findings suggest that DSI is not associated with a single problem, but that a complex of invasive problems threatens the social, mental and physical health of the DSI older adult.	Interactions during daily care, receiving information, having conversations about daily care and on daily LTC circumstances, might enable the DSI older adults to connect and live their lives as human beings, in connection with others. This form of support requires person-centered communication attitudes and LTC professionals' skills, especially of the nurses who provide daily care. address the participation and control-in-personal-space (CIPS) problems of DSI older adults, LTC professionals and researchers need to develop and test programs providing autonomy based and person-centered participation support such as, for example, the SMP-DSI program	NA
				Older adults with DSI perceived even more discrimination than those with either hearing or vision loss alone. Having DSI increased perceived discrimination about twice as much as being of non-White race. Perceived everyday discrimination is a multidomain construct, and DSI increased perceived discrimination across the domains tested in this analysis.However, the greatest increases were in the perception of being treated with less respect and the perception that people act as if the individual with DSI was "not smart."	Studies should seek to replicate the findings using objective measures of hearing and vision. More work is needed to address this discrimination, which may help to mitigate some of the negative consequences of vision and hearing loss among older adults.	NA

	Among males, having at least four ACEs was associated with the presence of SSI (OR = 2.91, 95 % CI = 1.09–7.74), as well as the progression from SSI to DSI (OR = 2.04, 95 % CI = 1.01–4.14) and maintained DSI (OR = 2.14, 95 % CI = 1.11–4.16). Among females, having at least four ACEs was associated with maintained DSI (OR = 2.19, 95 % CI = 1.04–4.58), but not the presence of SSI (OR = 0.74, 95 % CI = 0.31–1.77), DSI (OR = 1.78, 95 % CI = 0.75–4.24), and the progression from SSI to DSI (OR = 1.44, 95 % CI = 0.68–3.08).			This study revealed that, compared to participants without ACEs, experiencing four or more ACEs was positively associated with DSI, as well as the maintenance of DSI and progression from SSI to DSI over time. The association varied across different ACE types. Furthermore, linear dose-response associations of the number of ACEs with SVI and DSI were observed. Sex-stratified analysis showed that having at least four ACEs was specifically associated with DSI and its maintenance in females, while in males, it was linked with the presence of SSI in adulthood and the progression from SSI to DSI.	Future studies should consider amending questions and items related to ACEs based on cultural backgrounds. Thirdly, the inability to identify the included participants with early-onset SI (before ACEs) limits this research to establish a robust temporal sequence between ACEs and SI. Consequently, caution must be exercised in inferring a causal relationship between ACEs and SI, and more prospective studies are needed.	NA
In the unadjusted logistic regression analyses, auditory impairment, vision impairment, and DSI were associated with an increased risk of having clinically significant depressive and anxiety symptoms in the last month (compared to those without impairment). The odds ratio was highest for the DSI grouping (OR=5.99 for depression and OR=5.07 for anxiety). Sensory impairment was associated with clinically significant depressive symptoms: auditory impairment OR=1.43 (95% CI: 1.08–1.89), vision impairment OR=1.57 (95% CI: 1.04–2.37), and DSI OR=2.68 (95% CI: 1.45–4.96).	NA	Hispanic race and ethnicity was associated with visual impairment and DSI	NA	Our analyses of the association of auditory, vision, and DSI with clinically significant depressive and anxiety symptoms in a nationally representative sample of Medicare beneficiaries aged 65 years and older yielded findings congruent with our hypotheses. Consistent with prior literature, older adults with auditory impairment, vision impairment, and DSI were approximately 1.7 to 3.7 and 2.1 to 3.6 times more likely to have clinically significant depressive and anxiety symptoms, respectively. Those with DSI had the highest prevalence of depressive and anxiety symptoms. Additionally, even after accounting for sociodemographics, medical comorbidity, and functional impairment, the association with sensory impairment and depressive and anxiety symptoms persisted.	Clinicians working with older adults should consider screening for auditory and vision sensory impairments with their older adult patients with depression and/or anxiety. Likewise, clinicians should consider depression and anxiety screening with their older adult patients with auditory and/or vision impairments. Future research efforts could examine whether sensory impairment affects weak social ties	NA
Relative to those without any sensory impairments, hearing impairment only, vision impairment only, and DSI were all associated with a higher prevalence of SCCs after multivariable adjustment (see Table 2). DSI, notably, was associated with a higher prevalence of SCCs relative to those without sensory impairments (PR = 3.21; 95% confidence interval [CI] [2.83, 3.63]). There was an interaction between age group category and DSI category such that associations were attenuated among older age categories (e.g., PR = 0.51, p < .01 for hearing impairment and PR = 0.51, p < .01 for DSI among the 80–84 years age group; p < .001 for all DSI categories among the 85+ years age group; see Supplemental Material S2) after multivariable adjustment. There was no interaction between sex and DSI category	NA	NA	NA	DSI was cross-sectionally associated with a higher prevalence of subjective cognitive complaints (SCCs). One main finding is the observed higher prevalence of SCCs among those with DSI relative to those with each impairment alone and no impairment. Our analysis also alluded to an interaction between age and sensory impairment, which tended to weaken associations between DSI and SCCs in some later age groups.	Although these data indicate a cross-sectional association between multiple self-reported impairments and cognition and underscore the importance of analyzing concurrent impairments in the context of studies of cognition, future longitudinal and randomized studies are necessary to better elucidate the relationship	NA
After adjusting for covariates related to dementia, the OR for single-domain MCI was significantly higher in the VI group (OR: 1.31; 95% CI: 1.06–1.61), and the OR for multiple-domain MCI was significantly higher in the DSI group (OR: 1.58; 95% CI: 1.10–2.29) compared to other groups. Functional decline in processing speed was significantly greater in the DSI group than in the NSI group (OR: 2.03, 95% CI: 1.42–2.91).	NA	NA	NA	Participants with DSI exhibited functional decline in the cognitive domains of executive function and processing speed. Subjective hearing and visual impairment is associated with cognitive decline, and there is increasing need to establish appropriate treatment and preventive measures for persons with DSI, which is expected to increase as the population ages. In the present study, the group with DSI had a history of diabetes and heart disease. This study also showed that the DSI group exhibited a higher odds ratio for multiple-domain MCI, similar to the findings of previous studies. Furthermore, in relation to cognitive domains, the DSI showed a high OR for functional decline in executive function and processing speed, supporting the cognitive load hypothesis, which suggests that reduced processing of sensory information is associated with reduced cognitive function.	Early diagnosis and treatment of subjective symptoms related to vision may lead to the maintenance and improvement of cognitive function.	NA
An examination of the diversity of specific activities at baseline showed that those with DSIs had significantly lower instrumental, cognitive, social, and productive activities than did those with NSIs.	NA	NA	NA	This cross-sectional analysis indicated that those with DSI had a narrower life space than those with NSI and were subject to various activity limitations, including instrumental, cognitive, social, and pro- ductive activities. Furthermore, the longitudinal analysis suggested that those with DSI were associated with a narrower life space after two years. Maintaining an active lifestyle and life space in older adults is a protective factor against dementia; therefore, appropriate assessment and treatment of sensory impairments, such as HI and VI, is considered necessary.	Furthermore, there are various causes of reduced mobility in life space, and we cannot deny the possibility in this study of the impact of unmeasured factors, such as new-onset stroke, Parkinson's disease, or dementia during the 2-year follow-up period; furthermore, the causal relationship with DSI is unknown. A longer follow-up study from middle-aged patients is necessary to confirm the association reported in this study.	
as anticipated, VI and DSI were associated with cognitive functioning, but unexpectedly, HI was not.	NA		NA	DSI was associated with social isolation longitudinally, but not cross-sectionally. One interpretation of this finding is that DSI does not have an immediate impact on social isolation, but over time it may start to impact connection with others in important ways [48]. Social isolation can have profound negative impacts on older individuals' (ages 65+) health and is associated with increased risk for anxiety, depression, and dementia [	We recommend future research on the qualitative nature of family support among Hispanic families as it relates to living with sensory loss. Future measures of social isolation could include measures of social activities that are relevant to those of Hispanic ethnicity, e.g., going on picnics, attending dances, visiting family, etc.	NA
NA	NA	NA	NA	There are multiple reasons why older adults in rural areas have higher prevalence of DSI, loneliness, and social isolation than their urban counterparts.urprisingly, older adults with DSI are at a compounded risk of loneliness compared with those with HI only or VI only in rural areas, but not in urban areas.	NA	
Individuals with DSI generally had comorbidities of CCI score 1 or higher: CCI score 1 or 2 (n = 934; 30.6%), CCI score 3 or 4 (n = 830; 27.2%), and CCI score 5 or high (n = 851; 27.9%) (Table 1). The prevalence of hospitalization in DSI group was 31.7% (969/3,055), the highest among all four groups.mong beneficiaries with DSI, all models revealed a significantly higher odds of inpatient stay (unadjusted OR = 2.66; 95% CI = 2.46–2.87; age- adjusted OR = 2.05; 95% CI = 1.90–2.22; demographic- adjusted OR = 2.04; 95% CI = 1.88–2.20; fully adjusted OR = 1.41; 95% CI = 1.30–1.54), compared with bene- ficiaries with NSI.	NA	NA	NA	This study found that in all beneficiaries of the 2015 California Medicare population, patients with DSI had a 41% increase in the adjusted odds of at least one hospitalization compared to beneficiaries with NSI. Patients with DSI had a higher crude risk of hospitaliza- tion than patients with VIO, HIO, or NSI.	Further studies are indicated to find out potential rea- sons of this relationship and to develop strategies to prevent or delay DSI, which could improve patient out- comes and reduce burdens for the healthcare system	
In model 1, after adjusting for socio-demographic and other potentially confounding variables, VL and DSL were significantly associated with depression at baseline. Older adults with DSL had a higher odds ratio (OR=2.14, 95%CI: 1.77-2.58) compared to those with no sensory loss, while the odds ratio for VL was 1.46 (95%CI: 1.19-1.79). Moreover, baseline VL (OR=1.60, 95%CI: 1.16-2.22) and DSL (OR=1.78, 95%CI: 1.32-2.41) were associated with incident depression over four years, while there was no statistical significance between HL and incident depression. After introducing interactions, VL (OR=1.49, 95%CI: 1.13-1.97) and DSL (OR=2.25, 95%CI: 1.75-2.90) were associ- ated with depression at baseline. Over four years, both VL (OR=1.66, 95%CI: 1.16-2.21) and DSL (OR=1.60, 95%CI: 1.32-2.40) were still associated with depression. I	NA	NA	NA	The cross-sectional results showed that older adults with VL and DSL were more likely to have depressive symptoms compared to those with no sensory loss. Furthermore, VL and DSL were associated with incident depression over four years in those with no depression at baseline, whereas HL was not related to onset or incident depression. Moreover, interactions between all types of sensory loss and social activities were not significant. Considering the results in our sample for HL and VL separately, this finding suggests that older adults with VL and DSL may benefit from mental health intervention programs in the future.	NA	
Among respondents with SI, the DSI group had the highest mean depressive symptoms score (2.60), followed by the VI-only group (2.03) and the HI-only group (1.41).	NA	NA	NA	The results showed partial support for the main effects of SI and lack of social contact on number of depressive symptoms, and that the relationship between VI and DSI and number of depressive symptoms was weaker during the pan- demic as compared to before the pandemic. Those with DSI had the highest number of depressive symptoms.	We also explored whether gender or age moderated the relationship between SI and number of depressive symptoms. We found no statistically significant moderation effects. Future research should examine these possibilities with different data sources and measures.	

The greatest cognitive decline was seen in residents with DSI and social disengagement: 1.87 (95% CI = 1.24:2.51) point decrease in cognitive function over 1 year.	NA	NA	NA	Further, having social engagement is suggested to modify the association: we found that concurrent vision and hearing impairment was not associated with a greater cognitive decline when at least one behavior indicating social engagement was present.	NA
NA	NA	NA	NA	Furthermore, our study contributes evidence regarding the joint association of DSI and no involvement in activities with mortality.	NA
NA	NA	NA	NA	indings indicated that all cross-sectional associ- ations between VD, HD, and DSD and cognitive function operated through social isolation. However, longitudinally, only the associations of VD and DSD with cognitive function appeared to be significantly mediated by social isolation.Like VD and HD, DSD was associated with impaired cog- nitive functioning through social isolation cross-sectionally. DSD was also associated with learning/memory across 1 year through social isolation. Because VD and HD were in- dependently associated with impaired cognitive functioning, it was not surprising that DSD was also associated with impaired cognitive functioning.	NA
NA	Compared to other groups, older adults with DSI were more likely to be female (urban areas: 56.5%, p < .001; rural areas: 56.4%, p < .001), older (urban areas: 72.9 ± 8.4, p < .001; rural areas: 71.2 ± 7.4, p < .001), illiterate (urban areas: 38.4%, p < .001; rural areas: 47.9%, p < .001), single (urban areas: 26.0%, p < .001; rural areas: 23.9%, p < .001), and had a higher rate of unemploy- ment (urban areas: 57.5%, p < .001; rural areas: 64.2%, p < .001).	NA	NA	In this investiga- tion, we demonstrated that urban, but not rural, older adults with HI only or DSI had more institutional care willingness than those without SI. Unsurprisingly, we observed that urban older adults with HI only and DSI were more likely to show institutional care willingness than those without SI in this representative sample.	Future work should continue to focus on changes in the association between SI and willingness changes over time as well as mech- anisms that may contribute to the relationship.
older adults with DSI had a significantly higher level of depressive symptoms compared with those without SIs at baseline ($\beta = .07$, $p = .002$). The total effect of DSI at baseline on depressive symptoms at follow up was 0.453 (BCa 95% CI: 0.175–0.731). The indirect mediating effect of functional limitation was 0.060 (BCa: 95% CI: 0.031–0.094). Functional limitation partially mediated the relation- ship between DSI and depressive symptoms, which explained 13.25% of this relationship. Furthermore, analysis of the model before handling missing data also produced comparable results. For example, the total indirect effect of DSI on depressive symptoms was $\beta = .060$ ($p < .05$) or 11.19% of the total effect	NA	NA	NA	Results of this study revealed that older adults with DSI at baseline were significantly associated with a higher level of depressive symptoms at 3 year follow up, and functional limitation played a partially mediating role in such a relationship. Interventions should be focused on addressing DSI and functional limitation to mitigate depressive symptoms.	our study did not find a significant association between HI or VI and depressive symptoms, respectively, but observed a longitudinal association between DSI and increased depressive symptoms, which implies that future studies should shed more light on DSI.
**46**	**10**	**2**	**0**		
46					
21					
10					
9					
6					
6					
5					
3					
2					
1					
0					
0					
0					
